# Incidence and influential factors in pulp necrosis and periapical pathosis following indirect restorations: a systematic review and meta-analysis

**DOI:** 10.1186/s12903-023-02826-1

**Published:** 2023-04-02

**Authors:** Kholod Khalil Al-Manei, Shuruq Alzaidi, Ghadah Almalki, Khaled Al-Manei, Nabeel Almotairy

**Affiliations:** 1 Division of Endodontics, Department of Restorative Dental Science, College of Dentistry, King Saud University, Riyadh, 11545 Saudi Arabia; 2 College of Dentistry, King Saud University, Riyadh, 11545 Saudi Arabia; 3 Division of Oral Diagnostics and Rehabilitation, Department of Dental Medicine, Karolinska Institute, Alfred Nobels Allé 8, 141 04 Huddinge, Stockholm County, Sweden; 4 Department of Orthodontics and Pediatric Dentistry, College of Dentistry, Qassim University, Buraidah, 52571 Saudi Arabia

**Keywords:** Crown, Fixed partial denture, Periapical periodontitis, Pulp necrosis, Vital teeth

## Abstract

**Background:**

Restoring vital teeth with indirect restorations may threaten dental pulp integrity. However, the incidence of and influential factors on pulp necrosis and periapical pathosis in such teeth are still unknown. Therefore, this systematic review and meta-analysis aimed to investigate the incidence of and influential factors on pulp necrosis and periapical pathosis of vital teeth following indirect restorations.

**Methods:**

The search was conducted in five databases, using MEDLINE via PubMed, Web of Science, EMBASE, CINAHL, and Cochrane Library. Eligible clinical trials and cohort studies were included. The risk of bias was assessed using Joanna Briggs Institute’s critical appraisal tool and Newcastle–Ottawa Scale. The overall incidences of pulp necrosis and periapical pathosis following indirect restorations were calculated using a random effects model. Subgroup meta-analyses were also performed to determine the potential influencing factors for pulp necrosis and periapical pathosis. The certainty of the evidence was assessed using the GRADE tool.

**Results:**

A total of 5,814 studies were identified, of which 37 were included in the meta-analysis. The overall incidences of pulp necrosis and periapical pathosis following indirect restorations were determined to be 5.02% and 3.63%, respectively. All studies were assessed as having a moderate-low risk of bias. The incidence of pulp necrosis following indirect restorations increased when the pulp status was objectively assessed (thermal/electrical testing). The presence of pre-operative caries or restorations, treatment of anterior teeth, temporization for more than two weeks, and cementation with eugenol-free temporary cement, all increased this incidence. Final impression with polyether and permanent cementation with glass ionomer cement both increased the incidence of pulp necrosis. Longer follow-up periods (> 10 years) and treatment provided by undergraduate students or general practitioners were also factors that increased this incidence. On the other hand, the incidence of periapical pathosis increased when teeth were restored with fixed partial dentures, the bone level was < 35%, and the follow-up was > 10 years. The certainty of the evidence overall was assessed as low.

**Conclusions:**

Although the incidences of pulp necrosis and periapical pathosis following indirect restorations remain low, many factors affect these incidences that should thus be considered when planning indirect restorations on vital teeth.

**Database registration:**

PROSPERO (CRD42020218378).

**Supplementary Information:**

The online version contains supplementary material available at 10.1186/s12903-023-02826-1.

## Background

Indirect restorations are composed of artificial materials fabricated outside the oral cavity to restore severely damaged teeth [1, 2]. A broad range of indirect restorations is offered in dentistry, and these may be categorized into partial tooth coverage restorations such as inlays, onlays, and veneers, or full coverage restorations such as crowns or fixed partial dentures (FPD) [3]. Over time, the utilization of indirect restorations has increased in popularity, and the routine implementation of these restorations has become an integral part of the treatment modalities provided by most clinicians [4]. Estimates from the Adult Dental Health Survey in the United Kingdom show that 37% of adults with teeth had indirect restorations, with an average of three restorations per individual [5]. The increased utilization of indirect restorations is expected to allow teeth to be retained for a more extended period, yielding further increases in the maintenance and replacement of existing restorations.

The ultimate goals of indirect restoration are to restore teeth to proper form and function, minimize post-operative sensitivity, and preserve the vitality of the pulp [6, 7]. While tooth preparation and cementation are essential steps for indirect restoration, however, these procedures may induce various insults to the pulp and thus jeopardize pulp vitality. Previous studies have shown that tooth preparation disturbs the odontoblastic process and causes permanent damage to the odontoblasts [8–10]. Tooth preparation can also expose the dentinal tubules to the oral environment, creating a pathway for microorganisms to access dental pulp [11]. The desiccation from the air and marginal leakage of restorations may cause additional insults to the tooth pulp [12, 13]. The pulp health of teeth undergoing indirect restorations is also likely to be affected by preexisting cumulative insults from caries, periodontal diseases, trauma, or cracks [1, 2]. Once pulp vitality becomes dysfunctional, the pulp degenerates and may be invaded by microorganisms, eventually leading to total necrosis [14]. Depending on the host defense response and the degree of microbial virulence and their byproduct, untreated pulpal infection may also spread beyond the root apex, leading to periapical pathosis [15, 16].

Examining the scientific evidence for the biological consequences of dental pulp following indirect restorations does not only improve clinician knowledge of how to best diagnose and plan treatment but also allows clinicians to present realistic expectations to patients, as well as to assign the necessary time intervals for recall visits. It has been previously shown that the chance of endodontic diseases following indirect restoration procedures increases over time [17]. Within the past 25 years, a number of reports have also attempted to estimate the incidence of pulp necrosis and periapical pathosis following indirect restorations; however, these have yielded inconclusive findings [1, 2, 6]. Data from a recent systematic review and meta-analysis support the long-term success of the crown and FPD treatments on vital abutment teeth [18]. However, this review included only seven studies and lacked the assessment of various confounding factors such as cement type and examination method. Several other concerns have also been raised about that work, particularly in terms of the methodological approach, soundness, and the certainty of results [19]. The current study thus takes the form of a systematic review and meta-analysis and aims to overcome those limitations while investigating the following:

I. The overall incidence of pulp necrosis and periapical pathosis in vital teeth following indirect restorations.
II. The incidence of pulp necrosis and periapical pathosis among vital teeth restored with single-unit (veneer, inlay, onlay, and crown) versus multiple-unit (FPD) restorations.
III. Subgroup analyses of factors that may influence the incidence of pulp necrosis or periapical pathosis in vital teeth following indirect restorations.

## Methods

The protocol of this systematic review was registered prior to commencement in PROSPERO (CRD42020218378) and the review was conducted in accordance with the PRISMA-P guidelines.

### Inclusion and exclusion criteria

Studies were included on basis of the following criteria:

1. Study design: clinical trials (randomized and non-randomized) and cohort studies.
2. Participants: studies on humans’ permanent teeth.
3. Intervention: indirect dental restorations (single-unit and/ or FPD) in teeth diagnosed preoperatively with vital pulp and normal apical tissue.
4. Outcome: incidence of pulp necrosis and periapical pathosis.

Any study that did not satisfy at least one inclusion criterion was automatically excluded. Moreover, reviews, animal studies, editorials, and descriptive studies such as case reports and case series were not included in this study. Studies that evaluated the incidence of dentin hypersensitivity or pulpitis with indirect restorations were excluded. Studies that did not include an endodontic diagnosis of the teeth prior to indirect restoration placement or lack the follow-up were also excluded.

### Information sources

The search was conducted in October 2021 and updated in December 2022 by a senior researcher (NA) across five databases (MEDLINE via PubMed, Web of Science core collection, EMBASE, CINAHL, and Cochrane Library). The search strategy was built using appropriate free-text terms extracted from relevant studies using the PubReminer tool. These free-text terms were complemented with relevant MeSH/Emtree terms and thus truncated and/or combined with proximity operators. The search was completed using the built-in PubMed tools, then adapted for the other databases. To ensure the capture of all relevant studies, the database search was supplemented with a manual search of Google Scholar (first 300 results) and the Open Grey database (www.opengrey.eu), and forward and backward citations of eligible studies. No filters or restrictions were applied on the date or language of publication during searches. The full applied search strategy for this systematic review is thus shown in Supplementary file 1.

### Screening process for eligible studies

The search results obtained from the database and manual searches were checked for duplicates using the “check for duplicates” feature in Mendeley. The de-duplicated study list was then imported into a pre-established Excel template. Two independent researchers (SA and GA) screened the titles and abstracts of these studies and classified them as included, excluded, or undecided based on the inclusion and exclusion criteria. Indecision around any study’s inclusion was resolved by mutual discussion or by consulting senior researchers (KKA and KA). The full text of all potentially eligible studies was then critically examined to determine a final list of included studies, with any disagreements arising being again resolved by mutual discussion and consulting the senior researchers. The corresponding author for any study which needed further clarification was contacted.

### Quality assessment, data extraction, and certainty of evidence

The methodological quality and bias risk of the included studies were evaluated by two independent researchers (SA and GA) using the Joanna Briggs Institute (JBI) quality appraisal checklist and the Newcastle–Ottawa Quality Assessment Scale (NOS).

The JBI quality appraisal checklist was applied to assess the quality of all randomized clinical studies. This consists of thirteen items ranging from "true randomization used for assignment of participants to treatment groups" to "design appropriateness and deviation from standard randomized clinical trial design". Each item was answered “yes”, “no”, or “unclear”, and based on the percentage of "yes" answers, each study was classified into the appropriate group, with a low risk of bias at ≥ 75%, moderate risk of bias at ≥ 50%–< 75%, and high risk of bias at < 50%.

The NOS was used to assess the overall quality of all cohort studies. This considers three main domains: 1) sample selection, 2) comparability, and 3) exposure/outcome. The NOS adopted a star awards system, with each study awarded a maximum of one star for each item within sample selection and a maximum of two stars for comparability, based on the study design and analysis and outcome categories. The NOS thus allows generation of an overall quality score for each study out of 9, where scores of 1–3, 4–6, and 7–9 represent high, moderate, and low risk of bias, respectively. Any discrepancies during the quality assessment were resolved by consensus or by consulting senior researchers (KKA and KA). The overall evidence certainty was then assessed by applying the Grading of Recommendations Assessment, Development, and Evaluation (GRADE) tool (https://www.gradepro.org/).

Following the quality assessment, the relevant data obtained from the included studies were extracted by two independent researchers (SA and GA). This data included the authors, publication year, country/region, sample size, patient age and gender, medical history, study design, pre-operative tooth condition (e.g., intact, caries, previously restored), tooth type and location, pre-operative periodontal condition (e.g., probing depth, crown to root ratio, bone level), type of temporary cement, duration of temporization (≤ 2 weeks or > 2 weeks), type of impression material, type of permanent cement, type of indirect restoration (single-unit; FPD), materials used in the fabrication of indirect restorations, assessment method (objective: thermal and /or electrical pulp testing; subjective: clinical examination of signs and symptoms such as pain, tenderness, swelling, and/or sensitivity without specifying the use of thermal or electric pulp testing; periapical radiograph), level of practitioner training and expertise, post-treatment follow-up time (≤ 5 years, > 5–10 years, or > 10 years), and outcome measures (incidence of pulp necrosis and periapical pathosis).

### Statistical analysis

The kappa value for the inter-observer agreement was measured according to Cohen's kappa by calculating the probability of agreement minus the probability of random agreement divided by one minus the probability of random agreement. Publication bias was assessed by applying funnel plot, Egger’s, and Begg’s tests, while study heterogeneity was evaluated using Cochran Q (*Χ*^*2*^) and *I*^*2*^ statistics. According to Higgins et al*.* [20], study heterogeneity is considered low when *I*^*2*^ is between 0 and 25%. A fixed-effects model is used when study heterogeneity is low; otherwise, the data can be regarded as heterogenous, and a random-effects model is used. The data was treated as single arm to enhance the generalizability of the results. The meta-analysis was conducted to determine the overall incidences of pulp necrosis and periapical pathosis following indirect restorations at a 95% confidence interval (CI) using a random-effects model. The number of events (teeth diagnosed with pulp necrosis and/ or periapical pathosis following indirect restorations) out of the total number of vital teeth in each included study was calculated. The incidence of pulp necrosis and periapical pathosis among single-unit and FPD restorations was then analyzed. The incidence of pulp necrosis and periapical pathosis was also examined via subgroup analyses in order to determine the effects of the following factors: the method used to assess pulp status, type of temporary cement used, the time between temporary and permanent cementation, the impression material used, the fabrication material(s) used to create indirect restorations, the permanent cement used, the follow-up time and practitioner's training level. Statistical calculations were performed via MedCalc® Statistical Software (version 19.7.2, Ostend, Belgium; https://www.medcalc.org; 2021), with *P*-values of less than 0.05 considered statistically significant.

## Results

### Study selection, characteristics, and risk of bias

**Fig. 1:**
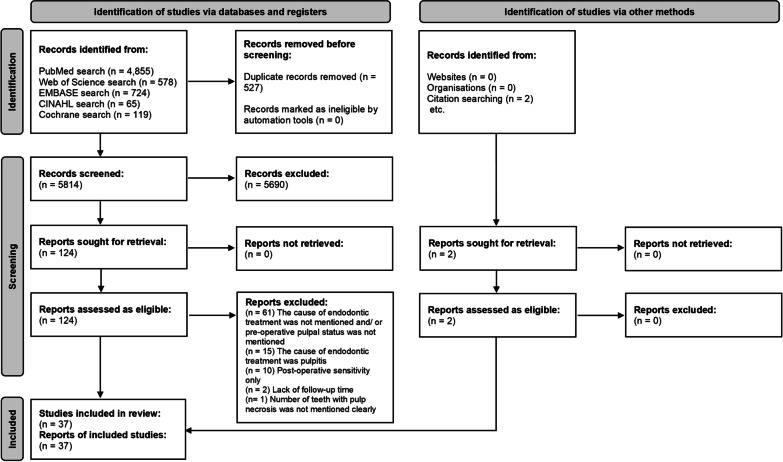
PRISMA flow chart for the study search, selection, and identification

Figure 1 shows the details of the study selection process within a PRISMA flow chart. Thirty-seven studies were included in the final analysis and eighty-nine were excluded (these studies are listed in Supplementary file 2). The total number of teeth included in this review was 11,615. Table 1 shows the baseline characteristics of the included studies.

**Table 1:** Baseline characteristics of the included studies.

Author, year, country, study design	Sample age; gender; medical history	Pre-operative pulpal and periapical status	Assessment method	Restoration type (single unit or FPD) and material	Impression material	Temporization cement and duration	Permanent cement	Clinical expertise	Follow-up time; PN; PP
Aziz et al., 2022 [21], Canada, Cohort	28–88 years; F:121, M:68; NA	Pulpal: Vital (55 teeth); Periapical: NA	Radiographic	Single-unit; Ceramic	Polyvinyl siloxane	NA; NA	Resin	Predoctoral students and trained dentists	> 5–10 years; 2; NA
Bergenholtz & Nyman [22], 1984, Sweden, Cohort	21–68 years; NA; NA	Pulpal: Vital (255 teeth); Periapical: NA	Objective	FPD; NA	NA	NA; NA	NA	NA	> 10 years; 38; 38
Chaar et al., 2020 [23], Germany, Cohort	37–70 years; F:23, M:16; NA	Pulpal: Vital (44 teeth); Periapical: Normal	Subjective	Single-unit; PFM	Polyether	NA; NA	GIC	NA	> 10 years; 1; NA
Cheung, 1991 [24], Hong Kong, Cohort	17–73 years; F:85, M:47; NA	Pulpal: Vital (73 teeth); Periapical: NA	Subjective	Single-unit; Gold, metal, porcelain	NA	NA; NA	NA	Students and general practitioners	≤ 5 years; 3; NA
Cheung et al., 2005 [1], China, Cohort	NA; NA; NA	Pulpal: Vital (199 teeth); Periapical: NA	Objective	Single-unit and FPD; PFM	NA	NA; NA	NA	NA	> 10 years; 44; NA
Denner et al., 2007 [25], Germany, RCT	22–65 years; F:22, M:38; NA	Pulpal: Vital (120 teeth); Periapical: NA	Objective	Single-unit and FPD; Metal or PFM	NA	Eugenol-based; NA	GIC or resin	NA	≤ 5 years; 2; NA
Derchi et al., 2019 [26], Germany, Cohort	23–65 years; F:17, M:13; NA	Pulpal: Vital (99 teeth); Periapical: Normal	Objective	Single-unit; Composite resin	Polyvinyl siloxane	NA; ≤ 2 weeks	Resin	General practitioners	> 10 years; 2; NA
Encke et al., 2009 [27], Germany, RCT	41.0–42.7 years; F:118, M:104; NA	Pulpal: Vital (138 teeth); Periapical: Normal	Objective	Single-unit; Ceramic or gold	Polyvinyl siloxane	Eugenol-free; NA	GIC	NA	≤ 5 years; 8; NA
Ericson et al., 1966 [28], Sweden, Cohort	20–79 years; F:178, M:94; NA	Pulpal: Vital (642 teeth); Periapical: Normal	Radiographic	FPD; NA	Compound	Eugenol-based; NA	Zinc phosphate	Students	≤ 5 years; 21; 21
Fayyad & AL-Rafee, 1996 [29], Saudi Arabia, Cohort	NA; NA; NA	Pulpal: Vital: (288 teeth); Periapical: NA	Radiographic	FPD; PFM, metal, or metal with acrylic facing	NA	NA; NA	NA	NA	> 5–10 years; 12; 12
Forrer et al., 2020 [30], Switzerland, Cohort	NA; F:52, M:30; NA	Pulpal: Vital (171 teeth); Periapical: NA	Objective	Single-unit and FPD; Ceramic or PFM	NA	NA; NA	GIC or resin	Predoctoral and postgraduate dentists	> 5–10 years; 6; 6
Hämmerle et al., 2000 [31], Switzerland, Cohort	29–84 years; F:70%, M:30%; NA	Pulpal: Vital: (120 teeth); Periapical: NA	Objective	FPD; PFM with gold frames	Polyvinyl siloxane or polysulfide	NA; NA	Zinc phosphate	Students and prosthodontists	> 10 years; 12; 3
Ioannidis & Bindl, 2016 [32], Switzerland, Cohort	52.6 ± 10.1 years; F:32, M:23; NA	Pulpal: Vital (57 teeth); Periapical: Normal	Objective	FPD; Ceramic with Y-TZP frameworks	Polyvinyl siloxane	Eugenol-free; NA	Resin	NA	> 5–10 years; 3; 1
Johnson et al., 1993 [33], United States of America, RCT	NA; NA; NA	Pulpal: Vital (214 teeth); Periapical: NA	Subjective	Single-unit; Gold or ceramic	Polyvinyl siloxane	Eugenol-based; NA	Zinc phosphate or GIC	Prosthodontists	≤ 5 years; 2; 2
Jokstad & Mjör, 1996 [34], Norway, Cohort	NA; NA; NA	Pulpal: Vital (86 teeth); Periapical: NA	Objective	Single-unit and FPD; Gold-resin or PFM	NA	Eugenol-based; > 2 weeks	Zinc phosphate or GIC	General practitioners	> 5–10 years; 5; NA
Karlsson, 1986 [35], Sweden, Cohort	34–78 years; F:89, M:75; NA	Pulpal: Vital (641 teeth); Periapical: NA	Radiographic	FPD; Acrylic resin veneer, gold or porcelain bonded to gold	NA	NA; NA	NA	General practitioners	> 5–10 years; 64; 64
Khazin et al., 2021 [36], Malaysia, Cohort	23–79 years; F:31, M:23; NA	Pulpal: Vital (73 teeth); Periapical: Normal	Objective	Single-unit and FPD; Metal, PFM, or ceramic	Polyvinyl siloxane	NA; NA	GIC, RMGIC, resin	NA	≤ 5 years; 1; NA
Kontakiotis et al., 2015 [6], Greece, Cohort	33–62 years; NA; NA	Pulpal: Vital (120 teeth); Periapical: Normal	Objective	Single-unit; NA	NA	Eugenol-free; > 2 weeks	NA	Students	≤ 5 years; 11; NA
Lockard et al., 2002 [37], Florida, Cohort	NA; F:172, M:84; NA	Pulpal: Vital (1847 teeth); Periapical: Normal	Subjective	Single-unit and FPD; Ceramic, PFM or metal	Reversible hydrocolloid	Eugenol-based; > 2 weeks	Zinc phosphate, poly-carboxylate, orthoethoxybenzoic acid, resin, GIC	Prosthodontists	> 10 years; 22; 22
Lundgren et al., 2018 [38], Sweden, RCT	11–22 years; F:15, M:12; Amelogenesis imperfecta	Pulpal: Vital (227 teeth); Periapical: NA	Radiographic	Single-unit; Ceramic with zirconia framework	Polyether	Eugenol-based; > 2 weeks	Resin	General practitioners and prosthodontists	> 5–10 years; 7; 7
Lundqvist & Nilson, 1982 [39], Sweden, Cohort	21–90 years; F:22, M:18; NA	Pulpal: Vital (35 teeth); Periapical: NA	Objective	Single-unit and FPD; NA	NA	NA; NA	NA	Students	> 5–10 years; 5; 1
Olley et al., 2018 [4], United Kingdom, Cohort	49.11 ± 15.65 years; F:27, M:20; NA	Pulpal: Vital (143 teeth); Periapical: NA	Subjective	Single-unit; PFM, gold or ceramic	Polysulfide and alginate	NA; NA	GIC, zinc phosphate or resin	General practitioners	> 10 years; 6; 6
Piemjai and Adunphichet, 2022 [40], Thailand, Cohort	61.44 ± 10.81 years; F: 177, M: 83; NA	Pulpal: Vital (642 teeth); Periapical: NA	Objective	Single-unit and FPD; metal, PFM	NA	NA; NA	Zinc phosphate, polycarboxylate, GIC, Resin	Postgraduates	> 10 years; 13; NA
Rauch et al., 2017 [41], Germany, Cohort	46.5 ± 13.1 years; F:21, M:13; Healthy	Pulpal: Vital (17 teeth); Periapical: NA	Objective	Single-unit; Ceramic	NA	NA; NA	Resin	NA	> 5–10 years; 3; NA
Reich & Schierz, 2013 [42], Germany, Cohort	26.2–73.8 years; F:21, M:13; Healthy	Pulpal: Vital (24 teeth) Periapical: NA	Objective	Single-unit; Ceramic	NA	NA; NA	Resin	NA	≤ 5 years; 2; NA
Reichen-Graden & Lang, 1989 [43], Switzerland, Cohort	26–72 years; F:42, M:16; NA	Pulpal: Vital (134 teeth); Periapical: NA	Objective	FPD; Ceramic	NA	NA; NA	Zinc phosphate	Students	> 5–10 years; 5; 1
Rinke et al., 2013 [44], Germany, Cohort	26–76 years; F:36, M:39; NA	Pulpal: Vital (200 teeth); Periapical: NA	Objective	FPD; Ceramic with zirconia framework	Polyether	NA; NA	Zinc phosphate	Students	> 5–10 years; 4; NA
Rinke et al., 2015 [45], Germany, Cohort	25–74 years; F:39, M:29; NA	Pulpal: Vital (221 teeth); Periapical: NA	Subjective	Single-unit; Zirconia	Polyvinyl siloxane or polyether	NA; NA	Zinc phosphate or GIC	General practitioners	> 5–10 years; 19; NA
Rinke et al., 2020 [46], Germany, Cohort	48.9 ± 12, 9 years; F:45, M:24; NA	Pulpal: Vital (81 teeth); Periapical: NA	Objective	FPD; Ceramic	NA	NA; NA	Resin	General practitioners	≤ 5 years; 1; NA
Scheibenbogen et al., 1998 [47], Germany, Cohort	23–58 years; NA; NA	Pulpal: Vital (71 teeth); Periapical: NA	Objective	Single-unit; Ceramic and composites	Polyether	Eugenol-free; ≤ 2 weeks	Resin	Students	≤ 5 years; 1; NA
Selz et al., 2014 [48], Germany, RCT	25–65 years; F:33, M:27; NA	Pulpal: Vital (149 teeth); Periapical: NA	Objective	Single-unit; Ceramic	Polyether	Eugenol-free; NA	Resin and GIC	Prosthodontists	≤ 5 years; 11; NA
Tinschert et al., 2008 [49], Germany, Cohort	20–58 years; F:27, M:19; NA	Pulpal: Vital (104 teeth); Periapical: Normal	Subjective	FPD; PFM	NA	Eugenol-free; ≤ 2 weeks	Zinc phosphate or Resin	Students and General practitioners	≤ 5 years; 3; NA
Uzgur et al., 2016 [50], Turkey, Cohort	21–73 years; F:214, M:310; NA	Pulpal: Vital (1633 teeth); Periapical: NA	Objective	FPD; PFM	NA	Eugenol-based; ≤ 2 weeks	Zinc polycarboxylate	Prosthodontists	≤ 5 years; 18; NA
Valderhaug et al., 1997 [17], Norway, Cohort	25–69 years; NA; NA	Pulpal: Vital (291 teeth); Periapical: NA	Subjective	Single-unit and FPD; Gold with acrylic resin	NA	Eugenol-based; NA	Zinc phosphate	Students	> 10 years; 30; 4
Walther, 1995 [51], Germany, Cohort	19–88 years; NA; NA	Pulpal: Vital (1983 teeth); Periapical: NA	Objective	Single-unit; NA	Polyether	Eugenol-based; NA	NA	NA	≤ 5 years; 124; NA
Wolleb et al., 2012 [52], Switzerland, Cohort	34–84 years; F:28, M:17; 22% smokers, 11% diabetic patients	Pulpal: Vital (311 teeth); Periapical: NA	Objective	Single-unit and FPD; PFM or ceramic	Polyether	NA; NA	GIC or resin	Students	> 5–10 years; 9; 9
Zitzmann et al., 2021 [53], Switzerland, Cohort	13–80 years; F: 39, M: 32; NA	Pulpal: Vital (107 teeth); Periapical: NA	Objective	FPD; metal or ceramic	NA	NA; NA	Resin	Postgraduate and prosthodontists	≤ 5 years; 1; NA

Abbreviations according to their first appearance: *FPD* Fixed partial denture, *PN* Pulp necrosis, *PP* Periapical pathosis, *NA* Not applicable, *F* Female, *M* Male, *PFM* Porcelain fused to metal, *GIC* Glass ionomer cement, *RMGIC* Resin modified glass ionomer cement.

The incidence of pulp necrosis following indirect restoration was investigated in all included studies, while periapical pathosis was only reported in fifteen studies. Thirty-two of the included studies were cohort study designs and five were randomized clinical trials, and all of them were written in English. Based on the NOS scale, the methodological qualities of the cohort studies were judged as having moderate (16 studies) to low risk of bias (16 studies), while according to the JBI quality appraisal checklist, four randomized clinical trials scored as having a low risk of bias, and one randomized clinical trial was rated as having a moderate risk of bias. A detailed summary of these JBI and NOS assessments is provided in Supplementary files 3 and 4. The interobserver agreement for assessing study selection and risk of bias was rated as excellent (kappa 0.92) [54]. As an indication of publication biases, the funnel plot (Supplementary file 5) and the statistical results for both Begg's and Egger's tests showed no publication bias across any included studies (Begg’s test = 0.07, *P* = 0.555; Egger’s test = 1.74, *P* = 0.073).

### Meta-analysis

**Fig. 2:**
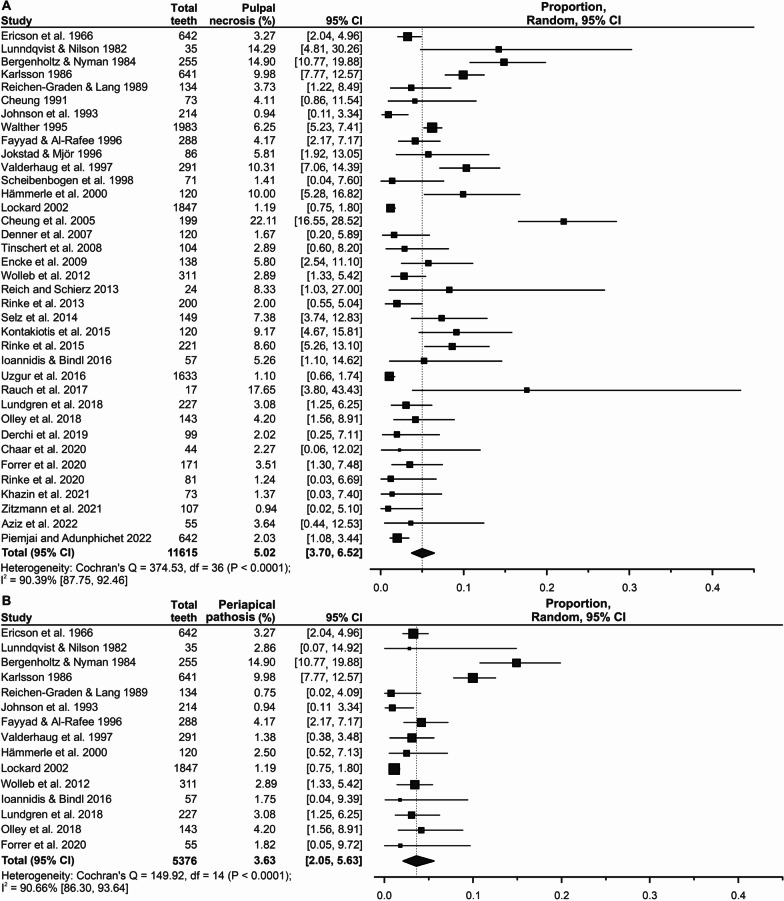
Random-effects meta-analysis for the overall incidence of pulp necrosis (**A**) and periapical pathosis (**B**) in vital teeth following indirect restorations. The black diamond indicates the cumulative incidence with a corresponding 95% confidence interval (CI)

**Fig. 3:**
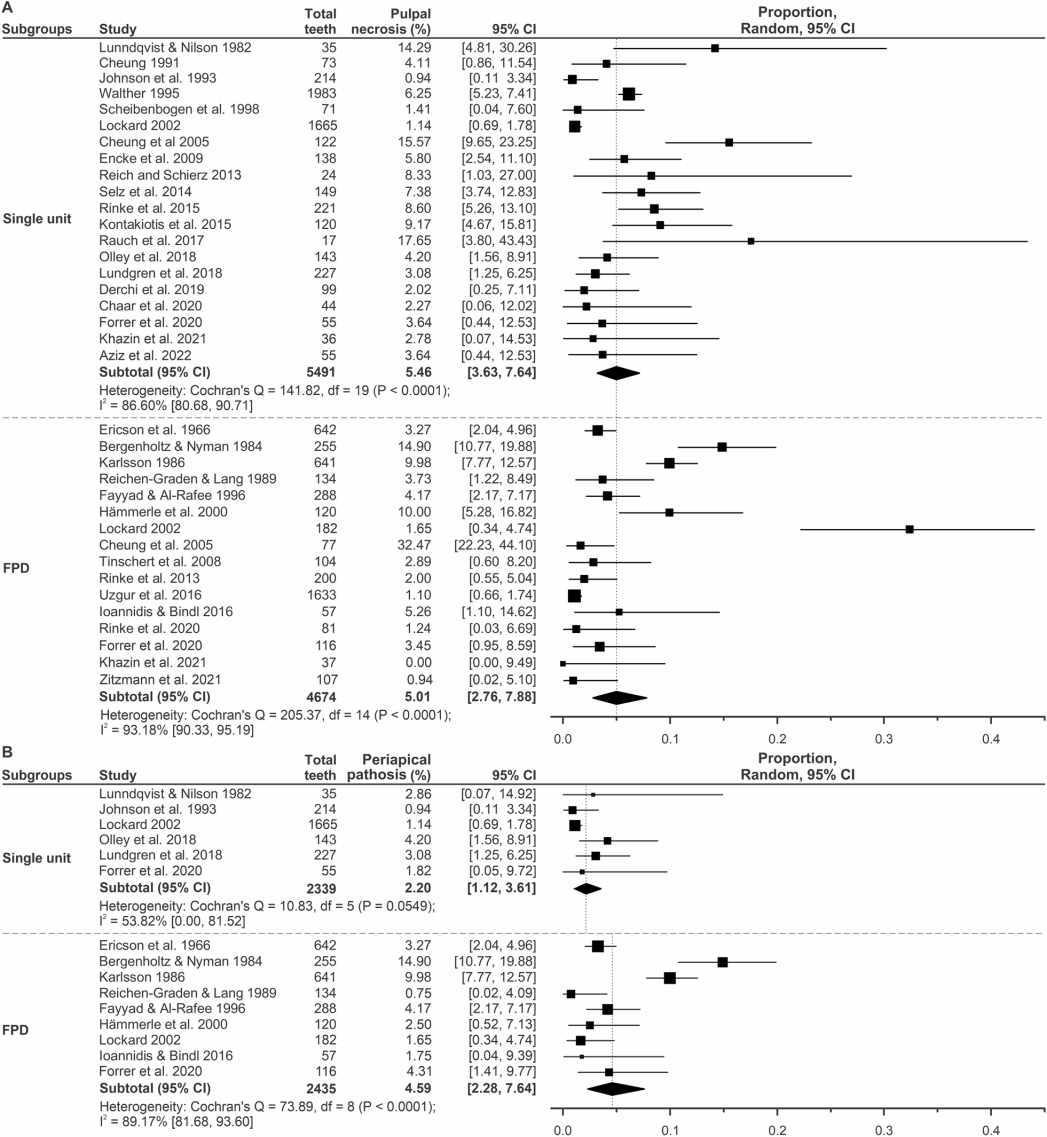
Random-effects meta-analysis for the incidence of pulp necrosis (**A**) and periapical pathosis (**B**) among the vital teeth restored with single-unit or FPD restorations. The black diamond indicates the cumulative incidence with a corresponding 95% confidence interval (CI)

The overall incidences of pulp necrosis and periapical pathosis following indirect restorations were 5.02% (Fig. 2A) and 3.63% (Fig. 2B), respectively. The incidence of pulp necrosis was relatively similar across those teeth restored with single-unit and those with FPD (single-unit = 5.46%; FPD = 5.01%; Fig. 3A). However, the incidence of periapical pathosis was higher in teeth restored with FPD than in teeth restored with single-unit restorations (FPD = 4.59%; single unit = 2.20%; Fig. 3B). A summary of the subgroup meta-analysis results for influential factors on the incidence of pulp necrosis and periapical pathosis is presented in Table 2.

**Table 2:** Summary of the subgroup meta-analysis results for the influential factors on the incidence of pulp necrosis and periapical pathosis in vital teeth following indirect restorations

Outcome	Subgroup analysis	Pulp necrosis				Periapical pathosis			
		**Total teeth**	**PN incidence %**	**CI (95%)**	***I***^***2***^***%***	**Total teeth**	**PP incidence %**	**CI (95%)**	***I***^***2***^***%***
Assessment method	Subjective	3181	3.91	1.86–6.66	88.07	–	–	–	–
	Objective	6581	5.66	3.71–7.99	91.14	–	–	–	–
	Radiographic	1853	4.95	2.43–8.29	86.57	–	–	–	–
Temporary cement	Eugenol-based	7043	3.34	1.66–5.58	94.43	–	–	–	–
	Eugenol-free	639	5.77	3.72–8.24	34.58	–	–	–	–
Temporization duration	≤ 2 weeks	1907	1.47	0.81–2.31	11.07	–	–	–	–
	> 2 weeks	2280	4.25	1.28–8.85	88.79	–	–	–	–
Impression material	Polyether	2985	4.02	2.47–5.92	69.04	–	–	–	–
	Polyvinyl siloxane	636	3.17	1.55–5.34	44.37	–	–	–	–
Restoration material	Ceramic	460	5.59	2.89–9.12	47.38	–	–	–	–
	PFM	1980	5.71	0.12–18.75	97.17	–	–	–	–
Permanent cement	Resin	738	3.37	1.90–5.23	32.02	–	–	–	–
	GIC	182	5.33	2.56–9.03	0	–	–	–	–
	Zinc phosphate	1387	4.92	3.85–6.20	85.09	–	–	–	–
Posttreatment follow-up	≤ 5 years	5532	3.53	2.10–5.31	86.66	856	2.26	0.59–4.98	73.4
	6–10 years	2443	5.53	3.74–7.64	73.95	1864	3.88	1.95–6.45	82.01
	> 10 years	3640	6.74	2.82–12.18	96.02	2656	4.05	0.89–9.36	94.86
Clinical expertise	Undergraduate students	1604	5.68	3.17–8.86	80.33	–	–	–	–
	General practitioners	2113	4.44	2.16–7.49	87.33	–	–	–	–
	Prosthodontists	3834	1.87	0.84–3.31	82.44	–	–	–	–

Abbreviations according to their first appearance: *PN* Pulp necrosis, *CI* Confidence interval, *I*^*2*^ Study heterogeneity, *PP* Periapical pathosis, *FPD* Fixed partial denture, *PFM* Porcelain-fused-to-metal, *GIC* Glass ionomer cement

### Assessment method

Eight studies used subjective methods [4, 17, 23, 24, 33, 37, 45, 49], twenty-four studies used objective methods [1, 6, 22, 25–27, 30–32, 34, 36, 39–44, 46–48, 50–53], and five studies used periapical radiographs [21, 28, 29, 35, 38] to evaluate the incidence of pulp necrosis following indirect restoration. The incidence of pulp necrosis was highest in teeth assessed using objective tools (5.66%), followed by periapical radiographs (4.95%), and subjective examination (3.91%).

### Temporary cement type and temporization period

Fifteen studies described the type of temporary cement (eugenol-based cement or eugenol-free cement) used for provisional restorations [6, 17, 25, 27, 28, 32–34, 37, 38, 47–51], and eight studies stated the time lapse between the temporization and permanent cementation (≤ 2 weeks or > 2 weeks) [6, 26, 34, 37, 38, 47, 49, 50]. Teeth temporized with eugenol-based cement and those with a short duration (≤ 2 weeks) before placement of indirect restorations had low incidences of pulp necrosis (eugenol-based cement = 3.34%; ≤ 2 weeks = 1.47%), whereas teeth temporized with eugenol-free cement or for longer periods (> 2 weeks) exhibited higher incidences of pulp necrosis (eugenol-free cement = 5.77%; > 2 weeks = 4.25%). Subgroup meta-analysis of the incidence of periapical pathosis could not be performed due to limited data.

### Impression material

Eighteen studies reported the type of impression materials used in the fabrication of indirect restorations [4, 21, 23, 26–28, 31–33, 36–38, 44, 45, 47, 48, 51, 52]; one study used compound material [28], another study used reversible hydrocolloid material [37], and three studies used more than one type of impression material [4, 31, 45]. The incidence of pulp necrosis was 3.27% when the compound material was used, but lower when reversible hydrocolloid material was used (1.19%). Thirteen studies used a single type of elastomeric impression material (polyether or polyvinyl siloxane) [21, 23, 26, 27, 32, 33, 36, 38, 44, 47, 48, 51, 52]. The incidence of pulp necrosis was higher when the impression was taken using polyether (4.02%) than where polyvinyl siloxane was used (3.17%). Subgroup meta-analysis of the incidence of periapical pathosis could not be performed due to limited data.

### Fabrication material

Five studies did not report the type of fabrication material used in the construction of indirect restorations [6, 22, 28, 39, 51], while twenty-two studies included different types of fabrication materials [4, 17, 24–27, 29–38, 40, 44, 45, 47, 52, 53]. A subgroup meta-analysis of the ten studies [1, 21, 23, 41–43, 46, 48–50] that used a single type of fabrication material (ceramic or porcelain-fused-to-metal [PFM]) was thus performed. The incidences of pulp necrosis among teeth restored with ceramic and PFM materials were quite similar (ceramic: 5.59%; PFM: 5.71%). Subgroup meta-analysis of the incidence of periapical pathosis could not be performed due to limited data.

### Permanent cement type

Twenty-nine studies reported the type of permanent cement used for indirect restorations [4, 17, 21, 23, 25–28, 30–34, 36–38, 40–50, 52, 53]. Of these, seventeen studies used a single type of permanent cement for cementation of indirect restorations [17, 21, 23, 26–28, 31, 32, 38, 41–44, 46, 47, 50, 53], and sixteen of these were included in the meta-analysis [17, 21, 23, 26–28, 31, 32, 38, 41–44, 46, 47, 53]. Teeth cemented with Glass Ionomer Cement (GIC) had the highest incidence of pulp necrosis (5.33%), followed by zinc phosphate cement (4.92%), and resin (3.37%). On the other hand, Uzgur et al*.* [50], in their study that featured a relatively large sample size found an extremely low incidence of pulp necrosis (1.1%) in teeth cemented permanently with zinc polycarboxylate. However, this study was excluded from the subgroup meta-analysis because it was the only study that used zinc polycarboxylate as permanent cement for indirect restorations. Subgroup meta-analysis of the incidence of periapical pathosis could not be performed due to limited data.

### Post-treatment follow-up period

The follow-up periods across the included studies were categorized into groups as ≤ 5 years, > 5–10 years, or > 10 years. A subgroup meta-analysis revealed that as the follow-up time increases, the incidence of pulp necrosis increases. Teeth with the longest follow-up times (> 10 years) following indirect restorations had a higher incidence of pulp necrosis than teeth after > 5–10 years or ≤ 5 years of follow-up time (> 10 years = 6.74%; > 5–10 years = 5.53%; ≤ 5 years = 3.53%). Similar results were also observed for the incidence of periapical pathosis, with teeth with > 10 years follow-up having the highest incidence (4.05%), followed by those at > 5–10 years (3.88%), and those at ≤ 5 years (2.26%) follow-up period.

### Practitioner training level

Twenty-nine studies reported the level of practitioner training during the placement of indirect restorations on vital teeth [4, 6, 17, 21, 24, 26, 28, 30, 31, 33–40, 42–50, 52, 53, 55]. Ten studies included more than one level of training without specifying the incidence of pulp necrosis for each training level [21, 24, 30, 31, 36, 38, 42, 49, 53, 55]. Nineteen studies ascribed the incidence of pulp necrosis to one level of training (undergraduate students or general practitioners or prosthodontists), and these were included in the meta-analysis [4, 6, 17, 26, 28, 33–35, 37, 39, 40, 43–48, 50, 52]. The incidence of pulp necrosis in teeth restored with indirect restorations by undergraduate students or general practitioners was higher than in teeth treated by prosthodontists (undergraduate students = 5.68%; general practitioners = 4.44%; prosthodontists = 1.87%). Subgroup meta-analysis of the incidence of periapical pathosis could not be performed due to limited data.

### Pre-operative tooth condition and tooth location

Eight studies reported the pre-operative tooth condition (intact, caries, previously restored and /or crowned, wear, fracture, and amelogenesis imperfecta) before the placement of indirect restorations [6, 17, 26, 33, 36, 38, 47, 52]. However, only one study investigated the effect of pre-operative tooth condition on the incidence of pulp necrosis following indirect restorations, and this showed teeth with pre-operative caries, fillings, or crowns had a higher incidence of pulp necrosis (13%) as compared to intact teeth (5%) [6]. The incidence of pulp necrosis in relation to the tooth location in the jaw (maxillary versus mandibular teeth and anterior versus posterior teeth) was reported in two studies [1, 6]. According to one, maxillary anterior teeth had the highest incidence of pulp necrosis following indirect restorations (30.2%), followed by maxillary posterior (23.7%), mandibular posterior (6.1%), and mandibular anterior (0%) teeth [1]. Conversely, the other study found that mandibular anterior teeth had the highest incidence of pulp necrosis (11.8%) as compared to maxillary anterior (9.4%), maxillary posterior (7.5%), and mandibular posterior (7.1%) teeth [6].

### Pre-operative periodontal condition

The influence of pre-operative periodontal condition (probing depth, crown to root ratio, bone level, furcation involvement, and tooth mobility) on the incidence of pulp necrosis and/or periapical pathosis were investigated in only a single study [36]. Bone level was the only periodontal factor found to influence the incidence of periapical pathosis. Specifically, teeth with a pre-operative bone level < 35% had a higher incidence of periapical pathosis (1.4%) than teeth with a bone level ≥ 35% (0%) [36].

### Certainty of evidence

The certainty of the evidence was rated as low for all outcome measures based on the assessment of the certainty parameters. A detailed summary of the certainty of the evidence is provided in Supplementary file 6.

## Discussion

Through the systematic search and meta-analysis, a strong body of evidence could be built regarding the number of vital teeth that may develop pulp necrosis and/or periapical pathosis after indirect restorations. This work is the first study that has assessed the incidence of pulp necrosis and periapical pathosis following indirect restorations of vital teeth as well as the potential factors that influence such incidence in this manner. Our findings revealed that the incidence of pulp necrosis and periapical pathosis following indirect restorations was relatively low. The incidence of pulp necrosis was found to be consistent for teeth restored via single-unit or FPD restorations. However, FPD-restored teeth had a higher incidence of periapical pathosis than teeth restored with single-unit restorations. According to subgroup meta-analyses, the incidence of pulp necrosis and periapical pathosis in vital teeth treated with indirect restorations appeared to be influenced by several factors.

As shown in this study, teeth restored with FPD have a higher incidence of periapical pathosis than teeth restored using single-unit restorations. FPD restorations are thought to generate higher occlusal forces than single-unit restorations, including vertical and transverse forces, potentially generating additional axial forces and stress gradients in the root, and supporting bone which may result in periapical radiographic changes. Consistent with the previous studies [1, 17], our finding revealed that the incidence of pulp necrosis among teeth restored with indirect restorations was influenced by the methods of assessing pulp status. Teeth assessed clinically by objective examination had a higher incidence of pulp necrosis than those assessed subjectively or by periapical radiographs. This could be related to the fact that the incidence of pulp necrosis may be underestimated when subjective and/or radiological assessment is used only as a diagnostic tool, as pulp necrosis can occur in the absence of radiographic changes or clinical signs and symptoms develop [56]. It should be noted that all the included studies used 2-dimensional periapical radiographs to assess periapical pathosis even though this method is widely acknowledged as being less accurate than cone-beam computed tomography (CBCT) [57, 58].

Another factor that influences the incidence of pulp necrosis following indirect restoration is the type of temporary cement used. Interestingly, teeth temporized with eugenol-containing cement demonstrated a lower incidence of pulp necrosis than teeth temporized with eugenol-free cement. Eugenol-containing cement has a wide-ranging disparity between toxicity to the pulp in absence of dentin protection relative to its safety when applied to dentin [59]. Although the biological effects of temporary cement are impacted by the thickness of dentin, several studies have highlighted the unique properties of eugenol-based cement, including antioxidant and sedative effects, lower dentin hypersensitivity, and the prevention of inflammatory responses in the pulp [59–61]. This could explain the lower incidence of pulp necrosis in teeth temporized with eugenol-containing cement. Meanwhile, the incidence of pulp necrosis is also influenced by the duration of temporization before permanent cementation. Our findings revealed that teeth temporized for more than two weeks had more pulp necrosis events than teeth temporized for two weeks or less. Results from previous studies showed that temporary cement has poor sealing abilities that may be associated with higher microleakage during long-term temporization [60, 62]. This drawback of temporary cement is further worsened in the presence of marginal gaps or ill-fitting provisional restorations, thus exposing the dentinal tubules to more irritants that may jeopardize pulp health.

As elastomeric impression materials (e.g., polyethers and polyvinyl siloxanes) have high accuracy and excellent properties, they are often used for indirect restorations [63]. The current findings revealed that the incidence of pulp necrosis was higher when polyether impression material was applied to teeth than polyvinyl siloxane. Generally, the shorter the contact between the tooth structure and impression materials, the less damage will be caused to the dental pulp [64]. However, polyethers have been found to be more toxic to fibroblast cells than polyvinyl siloxanes in human and animal studies [65–68]. Nonetheless, pulp necrosis does not appear to be impacted by the fabrication material applied to indirect restorations (i.e., PFM or ceramics). The similar incidence of pulp necrosis in these materials may be due to sufficient remaining dentinal thickness that protects the dental pulp [69, 70]. Our study also examined the impacts that permanent cement had on the incidence of pulp necrosis following indirect restorations. Although there is only one study that has examined polycarboxylate cement using a large sample, the cement has been found to have minimal impacts on dental pulp in comparison to other cement types [50]. This could be due to the biocompatibility properties of polycarboxylate cement which produces less fluoride (15–20%) than other cement types [71]. However, the incidence of pulp necrosis was highest with GIC cement due to the high acidity of GIC and the release of many fluoride ions which damage the dental pulp [71–73].

It is essential to implement long-term follow-up protocols after indirect restorations so that biological failures can be detected as early as possible. Our findings revealed that the incidence of pulp necrosis following indirect restorations increases as follow-up time is increased. From a clinical perspective, the increase in the incidence of pulp necrosis over time may be linked to the degree of irritation to the dental pulp from issues such as recurrent caries or trauma [6, 74]. Recent findings also revealed that elderly individuals have a greater risk of developing pulp necrosis [75]. Thus, it is reasonable to hypothesize that longer follow-ups enable aging-related changes to affect the pulp, which can result in fibrosis and reduced innervation. One of the most remarkable findings of this study is that practitioner training levels largely impact pulp necrosis following indirect restorations. The incidence of pulp necrosis was greater in teeth treated by general practitioners and undergraduate students than prosthodontists, which is in line with other research findings [76–79]. Moreover, experienced practitioners are more knowledgeable regarding treatment planning and have learned from previous failures [80]. Thus, it is important to consider the practitioner's competence level during indirect restoration procedures to ensure that the best clinical outcomes are achieved.

In this systematic review, only one study investigated the influence of pre-operative tooth conditions on the incidence of pulp necrosis. In that study, a higher incidence of pulp necrosis was observed in teeth with pre-operative caries, restorations, or previous crowns [6]. Teeth with intact enamel and healthy dentin-pulp complexes typically have protective mechanisms against external insults, whereas teeth that have been structurally compromised due to caries, trauma, or restorations may suffer from stress pulp conditions [69, 81]. Tooth preparation for indirect restoration may further irritate the pulp of compromised teeth, leading to pulpal death [82]. With regard to tooth type and location, a limited number of studies have investigated the incidence of pulp necrosis following indirect restorations [1, 6]. In the current study, maxillary and mandibular anterior teeth demonstrated a higher incidence of pulp necrosis than posterior teeth. This observation could be explained by the fact that anterior teeth are smaller than posterior teeth and removing the enamel and dentin during tooth preparation results in a thin dentinal structure near the pulp [6]. The clinical implication of this observation is particularly crucial in circumstances where the tooth's longitudinal axis must be modified during preparation. Rotated teeth, teeth with higher axial inclinations, and teeth with severe discoloration are examples of situations where additional reduction of tooth structure is required to obtain the proper angle of parallelism [1]. It is also noteworthy to mention that the distribution of teeth according to their types and locations in the relevant studies [1, 6] was not identical, meaning that the impacts of tooth type and location on pulp necrosis have not yet been examined.

The current study's findings showed limited evidence to support the influence of pre-operative bone level on the incidence of pulp necrosis and/or periapical pathosis in teeth restored with indirect restorations. In one study with a small sample size, periapical pathosis was more evident in teeth with a < 35% pre-operative bone level than in teeth with a ≥ 35% bone level [36]. Several histological studies have shown that bacterial plaque deposition on exposed root surfaces is associated with pathological changes in the adjacent pulp tissue, as this allows bacterial products to invade the pulp via exposed dentinal tubules and accessory canals [83, 84]. It is thus reasonable to assume that the greater alveolar bone loss, the greater the risk of bacterial irritants reaching and infecting the pulp; however, further longitudinal studies are required.

One of the strengths of the current systematic review is that it featured an extensive and comprehensive database search, thus capturing all available evidence on the research question with no restrictions on publication date or language. Furthermore, the nature of the included studies was focused on clinical trials and cohort studies, types of studies that provide a lot of evidence supporting observations of the incidences of pulp necrosis and periapical pathosis following indirect restorations. Another important strength was the use of subgroup analyses for those factors that influence the incidence of pulp necrosis and periapical pathosis following indirect restorations. These analyses undoubtedly added further insights into the potential impact of these factors on the incident of pulp necrosis and periapical pathosis. Finally, the GRADE tool was used to evaluate the certainty of the evidence in each measured outcome, revealing a low level of evidence.

However, the presence of high heterogeneity among the included studies is one of the current study's limitations. This could be due to the different clinical settings and methodologies used in included studies. Although a subgroup meta-analysis was performed, heterogeneity remained, and thus the results should be interpreted with caution. Furthermore, due to the limited number of studies reporting on pre-operative tooth conditions, tooth types, and per-operative periodontal conditions, a subgroup meta-analysis of these factors could not be performed. Another limitation is the small number of randomized clinical trials included, and more standardized clinical trials are required. A subgroup meta-analysis for partial versus full-coverage restorations was also not conducted, as the majority of the studies involved different designs for indirect restorations without specifying the incidence of pulp necrosis or periapical pathosis for each design.

## Conclusions

With a low-level of evidence, this study indicated that the incidence of pulp necrosis and periapical pathosis in vital teeth following indirect restorations was relatively low. Moreover, single-unit and multiple-unit restorations had the same level of impact on pulp necrosis, although single-unit restorations demonstrated a lower incidence of periapical pathosis than FPD. Many factors can influence pulp necrosis and periapical pathosis, although not all have been examined in this work. Thus, these factors must be carefully considered when planning indirect restorations.

## Supplementary Information

**Additional file 1: Supplementary file 1.** Database search strategy.

**Additional file 2: Supplementary file 2.** list of excluded articles.

**Additional file 3: Supplementary file 3.** Quality assessment of the included RCT studies.

**Additional file 4: Supplementary file 4.** Quality assessment of the included cohort studies.

**Additional file 5: Supplementary file 5.** Funnel plots.

**Additional file 6: Supplementary File 6.** GRADE assessment.

## Data Availability

The data used to support the findings of this study are available within the article and its supplementary files.
