# Supplementary material for: Incidence and influential factors in pulp necrosis and periapical pathosis following indirect restorations: a systematic review and meta-analysis
Source: BMC Oral Health. 2023 Apr 2;23:195. doi: 10.1186/s12903-023-02826-1 (PMC10069144; doi:10.1186/s12903-023-02826-1)
Supplement: Supplementary file 1 — Additional file 1: Supplementary file 1. Database search strategy. [file 12903_2023_2826_MOESM1_ESM.pdf]

**Supplementary file 1:** Database search strategy.

| PubMed | Results |
| --- | --- |
| (pulpal necrosis[Title/Abstract] OR pulpal health[Title/Abstract] OR pulpal complication[Title/Abstract] OR endodontic complication[Title/Abstract] OR periapical pathosis[Title/Abstract] OR periapical lesion[Title/Abstract] OR apical status[Title/Abstract] OR pulp* status[Title/Abstract] OR apical periodontitis[Title/Abstract]) OR (Root Canal Therapy / adverse effects* OR Prosthodontic / adverse effects* OR Dental Pulp Necrosis / epidemiology* OR Dental Pulp Necrosis / etiology OR Dental Restoration Failure OR Crowns / adverse effects* OR Cementation OR Cementation / adverse effects* OR Acid Etching OR Dental / adverse effects[MeSH Terms]) AND (bridge[Title/Abstract] OR crown[Title/Abstract] OR crown preparation[Title/Abstract] OR metal-ceramic crown[Title/Abstract] OR fixed prosthodontic[Title/Abstract] OR abutment teeth[Title/Abstract] OR vital abutment teeth[Title/Abstract] OR complete coverage restoration[Title/Abstract] OR full-coverage restoration[Title/Abstract] OR fixed partial denture[Title/Abstract] OR indirect restoration[Title/Abstract] OR indirect coronal restoration[Title/Abstract] OR fixed restoration[Title/Abstract] OR onlay[Title/Abstract] OR inlay[Title/Abstract]) | 4,855 |

| Web of Science Core Collection | Results |
| --- | --- |
| (TS=(bridge OR crown OR crown preparation OR metal-ceramic crown OR fixed prosthodontic OR abutment teeth OR vital abutment teeth OR complete coverage restoration OR full-coverage restoration OR fixed partial denture OR indirect restoration OR indirect coronal restoration OR fixed restoration OR onlay OR inlay) AND TS=(pulpal necrosis OR pulpal health OR pulpal complication OR endodontic complication OR ((periapical pathosis OR periapical lesion OR apical status OR pulp* status OR apical periodontitis Indexes=SCI-EXPANDED, SSCI, A&HCI, CPCI-S, CPCI-SSH, ESCI Timespan=All years | 578 |

| Embase | Results |
| --- | --- |
| tooth pulp':ti,ab OR ((pulp:ti,ab AND necrosis):ti,ab) OR ((pulp*:ti,ab AND health):ti,ab) OR ((pulp*:ti,ab')) AND complication):ti,ab) OR ((endodontic:ti,ab AND complication):ti,ab) OR 'tooth periapical disease':ti,ab OR periapical AND pathosis):ti,ab) OR ((periapical AND lesion):ti,ab) OR ((apical AND status):ti,ab) OR 'periapical)) tissue' OR ((pulp* AND status):ti,ab) OR ((apical AND 'periodontitis'):ti,ab)) AND (bridge:ti,ab OR 'tooth crown':ti,ab OR (crown:ti,ab AND preparation:ti,ab) OR ('metal ceramic':ti,ab AND crown:ti,ab) OR prosthodontic':ti,ab AND fixed:ti,ab) OR (fixed:ti,ab AND 'denture':ti,ab) OR 'denal procedure' OR ((vital:ti,ab') OR abutment:ti,ab) AND 'tooth':ti,ab) OR (complete:ti,ab AND coverage:ti,ab) OR 'full coverage':ti,ab OR partial:ti,ab AND denture:ti,ab) OR (indirect:ti,ab AND restoration:ti,ab) OR (coronal:ti,ab AND) ((restoration:ti,ab) OR (fixed:ti,ab AND 'dental restoration':ti,ab) OR onlay:ti,ab OR inlay:ti,ab | 724 |

| CINAHL | Results |
| --- | --- |
| TI ( pulpal necrosis OR pulpal health OR pulpal complication OR endodontic complication OR periapical) pathosis OR periapical lesion OR apical status OR pulp* status OR apical periodontitis ) OR MH ( Root Canal Therapy / adverse effects* OR Prosthodontic / adverse effects* OR Dental Pulp Necrosis / epidemiology* OR Dental Pulp Necrosis / etiology OR Dental Restoration Failure OR Crowns / adverse effects* OR Cementation OR Cementation / adverse effects* OR Acid Etching OR Dental / adverse effects )) AND (TI bridge OR crown OR crown preparation OR metal-ceramic crown OR fixed prosthodontic OR abutment teeth OR vital abutment teeth OR complete coverage restoration OR full-coverage restoration OR fixed partial denture OR indirect (restoration OR indirect coronal restoration OR fixed restoration OR onlay OR inlay | 65 |

| Cochrane Library | Results |
| --- | --- |
| bridge OR crown OR crown preparation OR metal-ceramic crown OR fixed prosthodontic OR abutment teeth) OR vital abutment teeth OR complete coverage restoration OR full-coverage restoration OR fixed partial denture OR indirect restoration OR indirect coronal restoration OR fixed restoration OR onlay OR | 119 |

inlay):ti,ab,kw AND (pulpal necrosis OR pulp* health OR pulp* complication OR endodontic complication OR periapical pathosis OR periapical lesion OR apical status OR pulp* status OR apical periodontitis):ti,ab,kw
