# Supplementary material for: Incidence and influential factors in pulp necrosis and periapical pathosis following indirect restorations: a systematic review and meta-analysis
Source: BMC Oral Health. 2023 Apr 2;23:195. doi: 10.1186/s12903-023-02826-1 (PMC10069144; doi:10.1186/s12903-023-02826-1)
Supplement: Supplementary file 2 — Additional file 2: Supplementary file 2. list of excluded articles. [file 12903_2023_2826_MOESM2_ESM.pdf]

Supplementary file 2: list of excluded articles

Excluded articles	Reason
Jackson et al. 1992 [1]	The cause of the endodontic treatment was pulpitis
Eckerbom 1993 [2]	Pre-operative pulpal status was not mentioned
Palmqvisl and Swartz 1993[3]	The cause of the endodontic treatment was not mentioned
Milleding et al. 1995 [4]	The cause of the endodontic treatment was pulpitis
Carlson and Yontchev 1996 [5]	The cause of the endodontic treatment was not mentioned
Decock et al. 1996 [6]	The cause of endodontic treatment was not mentioned, and pre-operative pulpal status was not mentioned
Sorensen et al. 1998 [7]	Post-operative sensitivity only
Saunders and Saunders 1998 [8]	Lack of follow up time
Burke et al. 1998 [9]	The cause of the endodontic treatment was not mentioned
Thordrup et al. 1999 [10]	Post-operative sensitivity
Studer et al. 2000 [11]	The cause of endodontic treatment was not mentioned
Thordrup et al. 2001 [12]	Post-operative sensitivity
Wenz et al. 2001 [13]	The cause of endodontic treatment was not mentioned, and Pre-operative pulpal status was not mentioned
Schulz et al. 2003 [14]	The cause of the endodontic treatment was not mentioned
Sjögren et al. 2004 [15]	The cause of endodontic treatment was not mentioned
Smales and Etemadi 2004 [16]	The cause of endodontic treatment was pulpitis
Kirkevang et al. 2004[17]	The cause of the endodontic treatment was not mentioned
Chai et al. 2005 [18]	The cause of endodontic treatment was not mentioned
Ohlmann et al. 2006 [19]	The cause of the endodontic treatment was not mentioned
Backer et al. 2006 [20]	The cause of endodontic treatment was not mentioned
Etemadi and Smales 2006 [21]	The cause of endodontic treatment was pulpitis
Heinemann et al. 2006 [22]	The cause of endodontic treatment was not mentioned, and Pre-operative pulpal status was not mentioned
Nickenig et al. 2006 [23]	The cause of endodontic treatment was not mentioned, and Pre-operative pulpal status was not mentioned
Fabianelli et al. 2006 [24]	Post-operative sensitivity
Eliasson et al. 2007 [25]	The cause of endodontic treatment was not mentioned

Signore et al. 2007 [26]	The cause of the endodontic treatment was pulpitis
Nickenig et al. 2008 [27]	Pre-operative pulpal status was not mentioned
Krämer et al. 2008 [28]	Post-operative sensitivity only
Crisp et al. 2008 [29]	The cause of the endodontic treatment was pulpitis
Jalalian et al. 2009 [30]	Post-operative sensitivity
Burke and Lucarotti 2009[31]	Pre-operative pulpal status was not mentioned
Behr et al. 2009 [32]	Pre-operative pulpal status was not mentioned
Frankenberger et al. 2009 [33]	The cause of endodontic treatment was pulpitis
Eschbach et al. 2009 [34]	Pre-operative pulpal status was not mentioned
Wolfart et al. 2009 [35]	Pre-operative pulpal status was not mentioned
Ortorp et al. 2009[36]	The cause of endodontic treatment was not mentioned
Roediger et al. 2010 [37]	Pre-operative pulpal status was not mentioned
Abou Tara et al. 2011 [38]	The cause of endodontic treatment was not mentioned, and Pre-operative pulpal status was not mentioned
Katsoulis et al. 2011 [39]	The cause of endodontic treatment was pulpitis
Sax et al. 2011 [40]	Pre-operative pulpal status was not mentioned
Crisp et al. 2012 [41]	The cause of the endodontic treatment was pulpitis
Ortorp et al. 2012 [42]	The cause of the endodontic treatment was not mentioned
Ohlmann et al. 2012 [43]	Pre-operative pulpal status was not mentioned, and the cause of the endodontic treatment was not mentioned
Jefferies et al. 2013 [44]	Post-operative sensitivity
Rinke et al. 2013 [45]	The cause of the endodontic treatment was not mentioned
Walton 2013 [46]	The cause of endodontic treatment was not mentioned
ryaKükrer 2014 [47]	The cause of the endodontic treatment was pulpitis
Dutta et al. 2014[48]	Lack of follow up time
Dawson et al. 2014[49]	Pre-operative pulpal status was not mentioned
Reich et al. 2014 [50]	The cause of endodontic treatment was pulpitis
Güncü et al. 2015 [51]	The cause of endodontic treatment was pulpitis
Seydler et al. 2015 [52]	The cause of the endodontic treatment was not mentioned
Lundgren et al. 2015 [53]	Pre-operative pulpal status was not mentioned

Huettig and Gehrke 2016 [54]	The cause of the endodontic treatment was not mentioned
Jacinta et al. 2016 [55]	Post-operative sensitivity
Bomicke et al. 2017 [56]	The cause of endodontic treatment was not mentioned, and Pre-operative pulpal status was not mentioned
Rathmann et al. 2017 [57]	The cause of endodontic treatment was pulpitis
Gunge et al. 2018 [58]	The cause of endodontic treatment was pulpitis
Rauch et al. 2018 [59]	The cause of the endodontic treatment was not mentioned
Collares et al. 2018 [60]	Pre-operative pulpal status was not mentioned, and the cause of the endodontic treatment was not mentioned
Gheorghe et al. 2019 [61]	Pre-operative pulpal status was not mentioned and lack of follow up time
Ashnagar et al. 2019 [62]	The cause of endodontic treatment was not mentioned, and Pre-operative pulpal status was not mentioned
passia et al. 2019 [63]	The cause of the endodontic treatment was not mentioned
Pastora et al. 2019 [64]	The cause of endodontic treatment was pulpitis
Yavorek et al. 2020 [65]	The cause of endodontic treatment was not mentioned
scutella et al. 2020 [66]	Pre-operative pulpal status was not mentioned
Habibi et al. 2020[67]	The cause of endodontic treatment was not mentioned
Petersen 2020 [68]	The cause of endodontic treatment was not mentioned
cagidiaco et al. 2020[69]	The cause of endodontic treatment was not mentioned
Alsterstål-Englund et al. 2021[70]	The cause of endodontic treatment was not mentioned
Fotiadou et al. 2021 [71]	Pre-operative pulpal status was not mentioned
Breemer et al. 2021 [72]	The cause of endodontic treatment was not mentioned
Bukmir et al. 2021[73]	Pre-operative pulpal status was not mentioned
Meirinhos et al. 2021 [74]	Pre-operative pulpal status was not mentioned
Petrauskiene and Haug 2021 [75]	Pre-operative pulpal status was not mentioned
Eltoukhy et al. 2021[76]	Post-operative sensitivity only
Abdulrhman et al. 2021[77]	The cause of endodontic treatment was not mentioned
Zürcher et al. 2021[78]	The cause of endodontic treatment was not mentioned
Sezgin et al. 2021[79]	Pre-operative pulpal status was not mentioned

Alenezi et al. 2022 [80]	Pre-operative pulpal status was not mentioned
Nilsson et al. 2022 [81]	The cause of endodontic treatment was not mentioned
Kusakabe et al. 2022 [82]	The cause of endodontic treatment was not mentioned
Won et al. 2022 [83]	Pre-operative pulpal status was not mentioned
Canatan et al. 2022 [84]	Post-operative sensitivity only
Hammoudi et al. 2022 [85]	Pre-operative pulpal status was not mentioned
Malament et al. 2022 [86]	The cause of endodontic treatment was not mentioned
Al-Qudah et al. 2022 [87]	Pre-operative pulpal status was not mentioned
Yoshino et al. 2022 [88]	Pre-operative pulpal status was not mentioned
Hawthan et al. 2022 [89]	Number of teeth with pulp necrosis was not mentioned clearly

1. Jackson, C.R., A.E. Skidmore, and R.T. Rice, *Pulpal evaluation of teeth restored with fixed prostheses.* J Prosthet Dent, 1992. **67**(3): p. 323-5.
2. Eckerbom, M., *Prevalence and technical standard of endodontic treatment in a Swedish population. A longitudinal study.* Swed Dent J Suppl, 1993. **93**: p. 1-45.
3. Palmqvist, S. and B. Swartz, *Artificial crowns and fixed partial dentures 18 to 23 years after placement.* Int J Prosthodont, 1993. **6**(3): p. 279-85.
4. Milleding, P., U. Ortengren, and S. Karlsson, *Ceramic inlay systems: some clinical aspects.* J Oral Rehabil, 1995. **22**(8): p. 571-80.
5. Carlson, B.R. and E. Yontchev, *Long-term observations of extensive fixed partial dentures on mandibular canine teeth.* J Oral Rehabil, 1996. **23**(3): p. 163-9.
6. Decock, V., et al., *18-year longitudinal study of cantilevered fixed restorations.* Int J Prosthodont, 1996. **9**(4): p. 331-40.
7. Sorensen, J.A., et al., *IPS Empress crown system: three-year clinical trial results.* J Calif Dent Assoc, 1998. **26**(2): p. 130-6.
8. Saunders, W.P. and E.M. Saunders, *Prevalence of periradicular periodontitis associated with crowned teeth in an adult Scottish subpopulation.* Br Dent J, 1998. **185**(3): p. 137-40.
9. Burke, F.J., A.J. Qualtrough, and N.H. Wilson, *A retrospective evaluation of a series of dentin-bonded ceramic crowns.* Quintessence Int, 1998. **29**(2): p. 103-6.
10. Thordrup, M., F. Isidor, and P. Hörsted-Bindslev, *A 3-year study of inlays milled from machinable ceramic blocks representing 2 different inlay systems.* Quintessence Int, 1999. **30**(12): p. 829-36.
11. Studer, S.P., et al., *Long-term survival estimates of cast gold inlays and onlays with their analysis of failures.* J Oral Rehabil, 2000. **27**(6): p. 461-72.
12. Thordrup, M., F. Isidor, and P. Hörsted-Bindslev, *A 5-year clinical study of indirect and direct resin composite and ceramic inlays.* Quintessence Int, 2001. **32**(3): p. 199-205.

13. Wenz, H.J., K. Hertrampf, and K.M. Lehmann, *Clinical longevity of removable partial dentures retained by telescopic crowns: outcome of the double crown with clearance fit.* Int J Prosthodont, 2001. **14**(3): p. 207-13.
14. Schulz, P., A. Johansson, and K. Arvidson, *A retrospective study of Mirage ceramic inlays over up to 9 years.* Int J Prosthodont, 2003. **16**(5): p. 510-4.
15. Sjögren, G., M. Molin, and J.W. van Dijken, *A 10-year prospective evaluation of CAD/CAM-manufactured (Cerec) ceramic inlays cemented with a chemically cured or dual-cured resin composite.* Int J Prosthodont, 2004. **17**(2): p. 241-6.
16. Smales, R.J. and S. Etemadi, *Survival of ceramic onlays placed with and without metal reinforcement.* J Prosthet Dent, 2004. **91**(6): p. 548-53.
17. Kirkevang, L.L., M. Vaeth, and A. Wenzel, *Tooth-specific risk indicators for apical periodontitis.* Oral Surg Oral Med Oral Pathol Oral Radiol Endod, 2004. **97**(6): p. 739-44.
18. Chai, J., et al., *Retrospective survival analysis of 3-unit fixed-fixed and 2-unit cantilevered fixed partial dentures.* J Oral Rehabil, 2005. **32**(10): p. 759-65.
19. Ohlmann, B., et al., *Clinical performance of posterior metal-free polymer crowns with and without fiber reinforcement: one-year results of a randomised clinical trial.* J Dent, 2006. **34**(10): p. 757-62.
20. De Backer, H., et al., *A 20-year retrospective survival study of fixed partial dentures.* Int J Prosthodont, 2006. **19**(2): p. 143-53.
21. Etemadi, S. and R.J. Smales, *Survival of resin-bonded porcelain veneer crowns placed with and without metal reinforcement.* J Dent, 2006. **34**(2): p. 139-45.
22. Heinemann, F., T. Mundt, and R. Biffar, *Retrospective evaluation of temporary cemented, tooth and implant supported fixed partial dentures.* J Craniomaxillofac Surg, 2006. **34 Suppl 2**: p. 86-90.
23. Nickenig, H.J., C. Schäfer, and H. Spiekermann, *Survival and complication rates of combined tooth-implant-supported fixed partial dentures.* Clin Oral Implants Res, 2006. **17**(5): p. 506-11.
24. Fabianelli, A., et al., *A clinical trial of Empress II porcelain inlays luted to vital teeth with a dual-curing adhesive system and a self-curing resin cement.* J Adhes Dent, 2006. **8**(6): p. 427-31.
25. Eliasson, A., C.F. Arnelund, and A. Johansson, *A clinical evaluation of cobalt-chromium metal-ceramic fixed partial dentures and crowns: A three- to seven-year retrospective study.* J Prosthet Dent, 2007. **98**(1): p. 6-16.
26. Signore, A., et al., *A 4- to 6-year retrospective clinical study of cracked teeth restored with bonded indirect resin composite onlays.* Int J Prosthodont, 2007. **20**(6): p. 609-16.
27. Nickenig, H.J., et al., *Survival and complication rates of combined tooth-implant-supported fixed and removable partial dentures.* Int J Prosthodont, 2008. **21**(2): p. 131-7.
28. Krämer, N., et al., *Totally bonded ceramic inlays and onlays after eight years.* J Adhes Dent, 2008. **10**(4): p. 307-14.
29. Crisp, R.J., et al., *A clinical evaluation of all-ceramic bridges placed in UK general dental practices: first-year results.* Br Dent J, 2008. **205**(9): p. 477-82.
30. Jalalian, E., N. Meraji, and M. Mirzaei, *A comparison of the efficacy of potassium nitrate and Gluma desensitizer in the reduction of hypersensitivity in teeth with full-crown preparations.* J Contemp Dent Pract, 2009. **10**(1): p. 66-73.

31. Burke, F.J. and P.S. Lucarotti, *Re-intervention on crowns: what comes next?* J Dent, 2009. **37**(1): p. 25-30.
32. Behr, M., et al., *Self-adhesive resin cement versus zinc phosphate luting material: a prospective clinical trial begun 2003.* Dent Mater, 2009. **25**(5): p. 601-4.
33. Frankenberger, R., et al., *Operator vs. material influence on clinical outcome of bonded ceramic inlays.* Dent Mater, 2009. **25**(8): p. 960-8.
34. Eschbach, S., et al., *Clinical evaluation of all-ceramic posterior three-unit FDPs made of In-Ceram Zirconia.* Int J Prosthodont, 2009. **22**(5): p. 490-2.
35. Wolfart, S., et al., *Clinical outcome of three-unit lithium-disilicate glass-ceramic fixed dental prostheses: up to 8 years results.* Dent Mater, 2009. **25**(9): p. e63-71.
36. Ortorp, A., M.L. Kihl, and G.E. Carlsson, *A 3-year retrospective and clinical follow-up study of zirconia single crowns performed in a private practice.* J Dent, 2009. **37**(9): p. 731-6.
37. Roediger, M., et al., *Prospective evaluation of zirconia posterior fixed partial dentures: four-year clinical results.* Int J Prosthodont, 2010. **23**(2): p. 141-8.
38. Abou Tara, M., et al., *Clinical outcome of metal-ceramic crowns fabricated with laser-sintering technology.* Int J Prosthodont, 2011. **24**(1): p. 46-8.
39. Katsoulis, J., et al., *Prosthetic rehabilitation and treatment outcome of partially edentulous patients with severe tooth wear: 3-years results.* J Dent, 2011. **39**(10): p. 662-71.
40. Sax, C., C.H. Hämmerle, and I. Sailer, *10-year clinical outcomes of fixed dental prostheses with zirconia frameworks.* Int J Comput Dent, 2011. **14**(3): p. 183-202.
41. Crisp, R.J., et al., *A clinical evaluation of all-ceramic bridges placed in patients attending UK general dental practices: three-year results.* Dent Mater, 2012. **28**(3): p. 229-36.
42. Ortorp, A., M.L. Kihl, and G.E. Carlsson, *A 5-year retrospective study of survival of zirconia single crowns fitted in a private clinical setting.* J Dent, 2012. **40**(6): p. 527-30.
43. Ohlmann, B., C. Eiffler, and P. Rammelsberg, *Clinical performance of all-ceramic cantilever fixed dental prostheses: results of a 2-year randomized pilot study.* Quintessence Int, 2012. **43**(8): p. 643-8.
44. Jefferies, S.R., et al., *A bioactive dental luting cement--its retentive properties and 3-year clinical findings.* Compend Contin Educ Dent, 2013. **34 Spec No 1**: p. 2-9.
45. Rinke, S., et al., *Practice-based clinical evaluation of metal-ceramic and zirconia molar crowns: 3-year results.* J Oral Rehabil, 2013. **40**(3): p. 228-37.
46. Walton, T.R., *The up to 25-year survival and clinical performance of 2,340 high gold-based metal-ceramic single crowns.* Int J Prosthodont, 2013. **26**(2): p. 151-60.
47. Kükrer, D., et al., *A prospective clinical study of ceromer inlays: results up to 53 months.* Int J Prosthodont, 2004. **17**(1): p. 17-23.
48. Dutta, A., F. Smith-Jack, and W.P. Saunders, *Prevalence of periradicular periodontitis in a Scottish subpopulation found on CBCT images.* Int Endod J, 2014. **47**(9): p. 854-63.
49. Dawson, V., et al., *Periapical status of non-root-filled teeth with resin composite, amalgam, or full crown restorations: a cross-sectional study of a Swedish adult population.* J Endod, 2014. **40**(9): p. 1303-8.
50. Reich, S., et al., *Three-unit CAD/CAM-generated lithium disilicate FDPs after a mean observation time of 46 months.* Clin Oral Investig, 2014. **18**(9): p. 2171-8.

51. Güncü, M.B., et al., *Zirconia-based crowns up to 5 years in function: a retrospective clinical study and evaluation of prosthetic restorations and failures.* Int J Prosthodont, 2015. **28**(2): p. 152-7.
52. Seydler, B. and M. Schmitter, *Clinical performance of two different CAD/CAM-fabricated ceramic crowns: 2-Year results.* J Prosthet Dent, 2015. **114**(2): p. 212-6.
53. Pousette Lundgren, G., et al., *A Randomized Controlled Trial of Crown Therapy in Young Individuals with Amelogenesis Imperfecta.* J Dent Res, 2015. **94**(8): p. 1041-7.
54. Huettig, F. and U.P. Gehrke, *Early complications and performance of 327 heat-pressed lithium disilicate crowns up to five years.* J Adv Prosthodont, 2016. **8**(3): p. 194-200.
55. Santos, M.J., et al., *Clinical evaluation of ceramic inlays and onlays fabricated with two systems: 12-year follow-up.* Clin Oral Investig, 2016. **20**(7): p. 1683-90.
56. Bömicke, W., et al., *Short-Term Prospective Clinical Evaluation of Monolithic and Partially Veneered Zirconia Single Crowns.* J Esthet Restor Dent, 2017. **29**(1): p. 22-30.
57. Rathmann, F., et al., *Veneered zirconia inlay-retained fixed dental prostheses: 10-Year results from a prospective clinical study.* J Dent, 2017. **64**: p. 68-72.
58. Gunge, H., et al., *Retrospective clinical evaluation of posterior monolithic zirconia restorations after 1 to 3.5 years of clinical service.* J Oral Sci, 2018. **60**(1): p. 154-158.
59. Rauch, A., et al., *Clinical survival of chair-side generated monolithic lithium disilicate crowns:10-year results.* Clin Oral Investig, 2018. **22**(4): p. 1763-1769.
60. Collares, K., et al., *A practice based longevity study on single-unit crowns.* J Dent, 2018. **74**: p. 43-48.
61. Anca Gabriela Gheorghe1, V.M., Sanda Mihaela Popescu3, Alexandra Carina Bănică1, Mihaela Ionescu4, Lelia Mihaela Gheorghiță5, Cristina Fărcaș- Berechet5, Oana Andreea Diaconu5, Mihaela Jana Țuculină5, Alina Iren Moraru6, *FREQUENCY OF ENDODONTIC TREATMENT AND PREVALENCE OF APICAL PERIODONTITIS IN ABUTMENT TEETH – A RADIOLOGICAL STUDY.* Romanian Journal of Oral Rehabilitation, 2019. **11**: p. 10.
62. Ashnagar, S., et al., *Long-term survival of structurally compromised tooth preserved with crown lengthening procedure and restorative treatment: A pilot retrospective analysis.* J Clin Periodontol, 2019. **46**(7): p. 751-757.
63. Passia, N., M.S. Chaar, and M. Kern, *Outcome of posterior fixed dental prostheses made from veneered zirconia over an observation period of up to 13 years.* J Dent, 2019. **86**: p. 126-129.
64. Serra-Pastor, B., et al., *Periodontal and prosthetic outcomes on teeth prepared with biologically oriented preparation technique: a 4-year follow-up prospective clinical study.* J Prosthodont Res, 2019. **63**(4): p. 415-420.
65. Yavorek, A., et al., *The Incidence of Root Canal Therapy after Full-Coverage Restorations: A 10-Year Retrospective Study.* J Endod, 2020. **46**(5): p. 605-610.
66. Scutella, F., et al., *Reliability of Chair-side Monolithic CAD-CAM Generated Lithium Disilicate Single Crowns with Knife- Edge Finish Line: Up to 5-Year Retrospective Analysis of Clinical Performance.* Eur J Prosthodont Restor Dent, 2020. **28**(2).
67. Habibi, Y., et al., *Three-year clinical performance of monolithic and partially veneered zirconia ceramic fixed partial dentures.* J Esthet Restor Dent, 2020. **32**(4): p. 395-402.
68. Brignardello-Petersen, R., *There seems to be a low risk of undergoing root canal treatment after a full-coverage crown.* J Am Dent Assoc, 2020. **151**(11): p. e112.

69. Ferrari Cagidiaco, E., et al., *A randomized controlled clinical trial of two types of lithium disilicate partial crowns.* Am J Dent, 2020. **33**(6): p. 291-295.
70. Alsterstål-Englund, H., et al., *A retrospective clinical evaluation of extensive tooth-supported fixed dental prostheses after 10 years.* J Prosthet Dent, 2021. **125**(1): p. 65-72.
71. Fotiadou, C., et al., *Longevity of lithium disilicate indirect restorations in posterior teeth prepared by undergraduate students: A retrospective study up to 8.5 years.* J Dent, 2021. **105**: p. 103569.
72. Van den Breemer, C.R.G., et al., *Prospective clinical evaluation of 765 partial glass-ceramic posterior restorations luted using photo-polymerized resin composite in conjunction with immediate dentin sealing.* Clin Oral Investig, 2021. **25**(3): p. 1463-1473.
73. Peršić Bukmir, R., et al., *Coronal Restoration as a Predictor of Periapical Disease in Non-Endodontically Treated Teeth.* Acta Stomatol Croat, 2021. **55**(1): p. 56-68.
74. Meirinhos, J., et al., *Prevalence of Lateral Radiolucency, Apical Root Resorption and Periapical Lesions in Portuguese Patients: A CBCT Cross-Sectional Study with a Worldwide Overview.* Eur Endod J, 2021. **6**(1): p. 56-71.
75. Zilinskaite-Petrauskiene, I. and S.R. Haug, *A Comparison of Endodontic Treatment Factors, Operator Difficulties, and Perceived Oral Health-related Quality of Life between Elderly and Young Patients.* J Endod, 2021.
76. Eltoukhy, R.I., et al., *Indirect Resin Composite Inlays Cemented with a Self-adhesive, Self-etch or a Conventional Resin Cement Luting Agent: A 5 Years Prospective Clinical Evaluation.* J Dent, 2021. **112**: p. 103740.
77. Abdulrahman, S., et al., *Evaluation of the clinical success of four different types of lithium disilicate ceramic restorations: a retrospective study.* BMC Oral Health, 2021. **21**(1): p. 625.
78. Zürcher, A.N., et al., *Clinical outcomes of tooth-supported leucite-reinforced glass-ceramic crowns after a follow-up time of 13-15 years.* J Dent, 2021. **111**: p. 103721.
79. Sezgin, G.P., et al., *Effect of prosthetic restorations and root canal fillings on periapical health in a selected patient group.* Br Dent J, 2021. **231**(2): p. 127-132.
80. Alenezi, A., et al., *Secondary caries in fixed dental prostheses: Long-term clinical evaluation.* Clin Exp Dent Res, 2022.
81. Nilsson, G., et al., *Loss of pulp vitality correlated with the duration of the interim restoration and the experience of the dentist: A retrospective study.* J Prosthet Dent, 2022.
82. Kusakabe, S., et al., *Clinical assessment of resin-coating technique applied to exposed dentin after crown preparation.* Dent Mater J, 2022. **41**(2): p. 226-229.
83. Won, K. and Y. Berlin-Broner, *Factors Associated with the Need for a Primary Endodontic Treatment after Single-Unit Crown Cementation: A Retrospective Case-Control Study.* J Endod, 2022. **48**(6): p. 730-735.
84. Canatan, S., F.D. Oz, and S. Bolay, *A randomized, controlled clinical evaluation of two resin cement systems in the adhesion of CAD/CAM-fabricated resin nanoceramic restorations: 18-month preliminary results.* J Esthet Restor Dent, 2022. **34**(7): p. 1005-1014.
85. Hammoudi, W., et al., *Long-term results of a randomized clinical trial of 2 types of ceramic crowns in participants with extensive tooth wear.* J Prosthet Dent, 2022. **127**(2): p. 248-257.

86. Malament, K.A., et al., *Incidence of endodontic therapy after complete or partial coverage glass-ceramic restorations with a follow-up time of over 30 years: A prospective clinical study.* J Prosthet Dent, 2022.
87. Al-Qudah, A., D.A. Jawad, and M. Jaradat, *Periapical Status of Non-Root-Filled Teeth With Amalgam, Composite, or Crown Restorations: A Cross-Sectional Study.* Int Dent J, 2022.
88. Yoshino, K., et al., *Survival Rate of 3-unit Fixed Partial Dentures Replacing First Molars: A Retrospective Cohort Study.* Bull Tokyo Dent Coll, 2022.
89. Hawthan, M., B.R. Chrcanovic, and C. Larsson, *Retrospective clinical study of tooth-supported single crowns: A multifactor analysis.* Eur J Oral Sci, 2022. **130**(4): p. e12871.
