# Supplementary material for: Incidence and influential factors in pulp necrosis and periapical pathosis following indirect restorations: a systematic review and meta-analysis
Source: BMC Oral Health. 2023 Apr 2;23:195. doi: 10.1186/s12903-023-02826-1 (PMC10069144; doi:10.1186/s12903-023-02826-1)
Supplement: Supplementary file 3 — Additional file 3: Supplementary file 3. Quality assessment of the included RCT studies. [file 12903_2023_2826_MOESM3_ESM.pdf]

Supplementary file 3: Quality assessment of the included RCT studies

#	Authors (year)	Johnson et al. (1993)	Denner et al. (2007)	Encke et al. (2009)	Selz et al. (2014)	Lundgren et al. (2018)
	**JBI critical appraisal checklist for RCT**					
**1**	Was true randomization used for assignment of participants to treatment groups?	1	1	1	1	1
**2**	Was allocation to treatment groups concealed?	1	0	0	0	0
**3**	Were treatment groups similar at the baseline?	1	1	1	1	1
**4**	Were participants blind to treatment assignment?	1	1	0	0	0
**5**	Were those delivering treatment blind to treatment assignment?	0	0	0	0	0
**6**	Were outcomes assessors blind to treatment assignment?	0	0	0	1	1
**7**	Were treatment groups treated identically other than the intervention of interest?	1	1	1	1	1
**8**	Was follow up complete and if not, were differences between groups in terms of their follow up adequately described and analyzed?	1	1	1	1	1
**9**	Were participants analyzed in the groups to which they were randomized?	1	1	1	1	1
**10**	Were outcomes measured in the same way for treatment groups?	1	1	1	1	1
**11**	Were outcomes measured in a reliable way?	1	1	1	1	1
**12**	Was appropriate statistical analysis used?	1	1	1	1	1
**13**	Was the trial design appropriate, and any deviations from the standard RCT design (individual randomization, parallel groups) accounted for in the conduct and analysis of the trial?	1	1	1	1	1
Total score (%): Low risk= 75%-100%<br>Moderate risk= 50%-74%<br>High risk < 50%		**85%**	**77%**	**69%**	**77%**	**77%**

Abbreviations;
JBI = Joanna Briggs Institute
RCT= Randomized Clinical Trial
Scores: 1 = Yes, 0 = No, 0 = Unclear
