# Supplementary material for: Incidence and influential factors in pulp necrosis and periapical pathosis following indirect restorations: a systematic review and meta-analysis
Source: BMC Oral Health. 2023 Apr 2;23:195. doi: 10.1186/s12903-023-02826-1 (PMC10069144; doi:10.1186/s12903-023-02826-1)
Supplement: Supplementary file 4 — Additional file 4: Supplementary file 4. Quality assessment of the included cohort studies. [file 12903_2023_2826_MOESM4_ESM.pdf]

Supplementary file 4: Quality assessment of the included cohort studies by Newcastle-Ottawa Scale

#	Authors (year)	Ericson et al. (1966)	Lundqvist & Nilson (1982)	Bergenholtz & Nyman (1984)	Karlsso (1986)	Reichen-Graden & Lang (1989)	Cheung (1991)	Walther (1995)	Fayyad & Al-Rafee (1996)	Jokstad and Mjör (1996)	Valderhaug et al. (1997)
	**NOS for cohort studies**										
	**Sample Selection Criteria (Only 1 Star)**										
1	Diagnosis of pulpal and/ or periapical status a) Clinical examination or radiographic with validated instrument b) Without clinical examination or radiograph or based on self-reports c) No description	b	*	b	b	b	b	*	b	b	*
2	Representativeness and selection of the patients receiving indirect restoration a) Patients with indirect restoration selected from a defined catchment area, in a defined hospital or clinic, health maintenance organization, communities or random sample b) Potential for selection biases or not satisfying requirements in part c) No description	*	*	*	*	*	*	*	*	*	*
	**Comparability of Cases on the Basis of the Design or Analysis (Possibility of 2 Stars)**										
1	Control for confounders a) The exposure of interest (pulpal necrosis or periapical pathosis) is adjusted for the one confounder b) The exposure of interest (pulpal necrosis or periapical pathosis) is adjusted for two or more confounders c) No description related to the adjustment analysis for confounding factors	**	c	*	*	*	*	**	*	**	**
	**Evaluation of Pulpal Necrosis and Periapical Pathosis (Possibility of 2 Stars)**										
1	Diagnosis of pulpal necrosis and/ or periapical pathosis a) Clinical examination reporting the use of clinical examination and/ or radiographic evaluation b) Satisfied requirements in part (a) and the examiner was blinded to tooth status and/ or report of observer agreement–kappa c) Based on self-reports or not satisfying requirements in part (a/b) d) No description	**	*	*	*	*	*	*	*	*	*
2	Was follow-up long enough for outcomes to occur (≥4 yrs) a) Yes b) No	*	*	*	*	*	b	*	*	*	*
3	Response rate a) Rate of sample loss ≤ 30% b) Rate of sample loss >30% c) Not stated	*	b	c	b	c	c	*	c	b	b
4	Assessment of the statistical test a) The statistical test used to analyze the data is clearly described and appropriate, and the measurement of the association is presented, including confidence intervals and/ or the probability level (p value) b) The statistical test is not appropriate, not described or incomplete.	b	*	b	*	*	*	*	b	*	*
Total Stars (9):	Low risk= 7-9<br>Moderate risk= 4-6<br>High risk= 1-3	**7**	**5**	**4**	**5**	**5**	**4**	**8**	**4**	**6**	**7**

Abbreviations;
NOS = Newcastle-Ottawa Scale

#	Authors (year)	Scheibenbogen et al. (1998)	Hämmerle et al. (2000)	Lockard (2002)	Cheung et al. (2005)	Tinschert et al. (2008)	Wolleb et al. (2012)	Reich & Schierz (2012)	Rinke et al. (2013)	Rinke et al. (2015)
	**NOS for cohort studies**									
**Sample Selection Criteria (Only 1 Star)**										
**1**	Diagnosis of pulpal and/ or periapical status a) Clinical examination or radiographic with validated instrument b) Without clinical examination or radiograph or based on self-reports c) No description	b	*	*	b	b	*	b	b	b
**2**	Representativeness and selection of the patients receiving indirect restoration a) Patients with indirect restoration selected from a defined catchment area, in a defined hospital or clinic, health maintenance organization, communities or random sample b) Potential for selection biases or not satisfying requirements in part c) No description	*	*	c	*	*	*	*	*	*
**Comparability of Cases on the Basis of the Design or Analysis (Possibility of 2 Stars)**										
**1**	Control for confounders a) The exposure of interest (pulpal necrosis or periapical pathosis) is adjusted for the one confounder b) The exposure of interest (pulpal necrosis or periapical pathosis) is adjusted for two or more confounders c) No description related to the adjustment analysis for confounding factors	**	**	**	**	**	**	**	**	**
**Evaluation of Pulpal Necrosis and Periapical Pathosis (Possibility of 2 Stars)**										
**1**	Diagnosis of pulpal necrosis and/ or periapical pathosis a) Clinical examination reporting the use of clinical examination and/ or radiographic evaluation b) Satisfied requirements in part (a) and the examiner was blinded to tooth status and/ or report of observer agreement–kappa c) Based on self-reports or not satisfying requirements in part (a/b) d) No description	**	*	*	**	c	*	*	**	*
**2**	Was follow-up long enough for outcomes to occur (≥4 yrs) a) Yes b) No	b	*	*	*	b	*	*	*	*
**3**	Response rate a) Rate of sample loss ≤ 30% b) Rate of sample loss >30% c) Not stated	*	b	c	c	*	*	*	*	*
**4**	Assessment of the statistical test a) The statistical test used to analyze the data is clearly described and appropriate, and the measurement of the association is presented, including confidence intervals and/ or the probability level (p value) b) The statistical test is not appropriate, not described or incomplete.	*	*	b	*	*	*	*	*	*
Total Stars (9): Low risk= 7-9; Moderate risk= 4-6; High risk= 1-3		**7**	**7**	**5**	**7**	**5**	**8**	**7**	**8**	**7**

Abbreviations;
NOS = Newcastle-Ottawa Scale

#	Authors (year)	Kontakiotis et al. (2015)	Uzgur et al. (2016)	Ioannidis & Bindl (2016)	Rauch et al. (2017)	Olley et al. (2018)	Derchi et al. (2019)	Rinke et al. (2020)	Chaar et al. (2020)	Forrer et al. (2020)	Khazin et al. (2021)
	**NOS for cohort studies**										
	**Sample Selection Criteria (Only 1 Star)**										
1	Diagnosis of pulpal and/ or periapical status a) Clinical examination or radiographic with validated instrument b) Without clinical examination or radiograph or based on self-reports c) No description	*	*	b	b	b	b	b	b	*	*
2	Representativeness and selection of the patients receiving indirect restoration a) Patients with indirect restoration selected from a defined catchment area, in a defined hospital or clinic, health maintenance organization, communities or random sample b) Potential for selection biases or not satisfying requirements in part c) No description	*	*	*	*	c	c	*	c	*	*
	**Comparability of Cases on the Basis of the Design or Analysis (Possibility of 2 Stars)**										
1	Control for confounders a) The exposure of interest (pulpal necrosis or periapical pathosis) is adjusted for the one confounder b) The exposure of interest (pulpal necrosis or periapical pathosis) is adjusted for two or more confounders c) No description related to the adjustment analysis for confounding factors	**	**	**	**	**	**	**	**	**	**
	**Evaluation of Pulpal Necrosis and Periapical Pathosis (Possibility of 2 Stars)**										
1	Diagnosis of pulpal necrosis and/ or periapical pathosis a) Clinical examination reporting the use of clinical examination and/ or radiographic evaluation b) Satisfied requirements in part (a) and the examiner was blinded to tooth status and/ or report of observer agreement–kappa c) Based on self-reports or not satisfying requirements in part (a/b) d) No description	*	*	*	**	*	*	*	*	*	**
2	Was follow-up long enough for outcomes to occur (≥4 yrs) a) Yes b) No	b	b	*	*	*	*	b	*	*	b
3	Response rate a) Rate of sample loss ≤ 30% b) Rate of sample loss >30% c) Not stated	c	c	*	*	c	*	*	c	*	*
4	Assessment of the statistical test a) The statistical test used to analyze the data is clearly described and appropriate, and the measurement of the association is presented, including confidence intervals and/ or the probability level (p value) b) The statistical test is not appropriate, not described or incomplete.	*	*	*	*	*	*	*	*	*	*
Total Stars (9):	Low risk= 7-9<br>Moderate risk= 4-6<br>High risk= 1-3	6	7	7	8	5	6	6	5	8	8

Abbreviations;
NOS = Newcastle-Ottawa Scale

#	Authors (year)	Zitzmann et al. (2021)	Aziz et al. (2022)	Piemjai and Adunphichet (2022)
	**NOS for cohort studies**			
	**Sample Selection Criteria (Only 1 Star)**			
**1**	Diagnosis of pulpal and/ or periapical status a) Clinical examination or radiographic with validated instrument b) Without clinical examination or radiograph or based on self-reports c) No description	*	*	*
**2**	Representativeness and selection of the patients receiving indirect restoration a) Patients with indirect restoration selected from a defined catchment area, in a defined hospital or clinic, health maintenance organization, communities or random sample b) Potential for selection biases or not satisfying requirements in part c) No description	*	*	*
	**Comparability of Cases on the Basis of the Design or Analysis (Possibility of 2 Stars)**			
**1**	Control for confounders a) The exposure of interest (pulpal necrosis or periapical pathosis) is adjusted for the one confounder b) The exposure of interest (pulpal necrosis or periapical pathosis) is adjusted for two or more confounders c) No description related to the adjustment analysis for confounding factors	*	*	*
	**Evaluation of Pulpal Necrosis and Periapical Pathosis (Possibility of 2 Stars)**			
**1**	Diagnosis of pulpal necrosis and/ or periapical pathosis a) Clinical examination reporting the use of clinical examination and/ or radiographic evaluation b) Satisfied requirements in part (a) and the examiner was blinded to tooth status and/ or report of observer agreement–kappa c) Based on self-reports or not satisfying requirements in part (a/b) d) No description	*	*	*
**2**	Was follow-up long enough for outcomes to occur (≥4 yrs) a) Yes b) No	b	*	*
**3**	Response rate a) Rate of sample loss ≤ 30% b) Rate of sample loss >30% c) Not stated	*	*	c
**4**	Assessment of the statistical test a) The statistical test used to analyze the data is clearly described and appropriate, and the measurement of the association is presented, including confidence intervals and/ or the probability level (p value) b) The statistical test is not appropriate, not described or incomplete.	*	*	*
Total Stars (9): Low risk= 7-9; Moderate risk= 4-6; High risk= 1-3		**6**	**7**	**6**
