# Supplementary material for: Incidence and influential factors in pulp necrosis and periapical pathosis following indirect restorations: a systematic review and meta-analysis
Source: BMC Oral Health. 2023 Apr 2;23:195. doi: 10.1186/s12903-023-02826-1 (PMC10069144; doi:10.1186/s12903-023-02826-1)

Supplementary file 5: Funnel plots

Assessment method_Pulp ncrosis

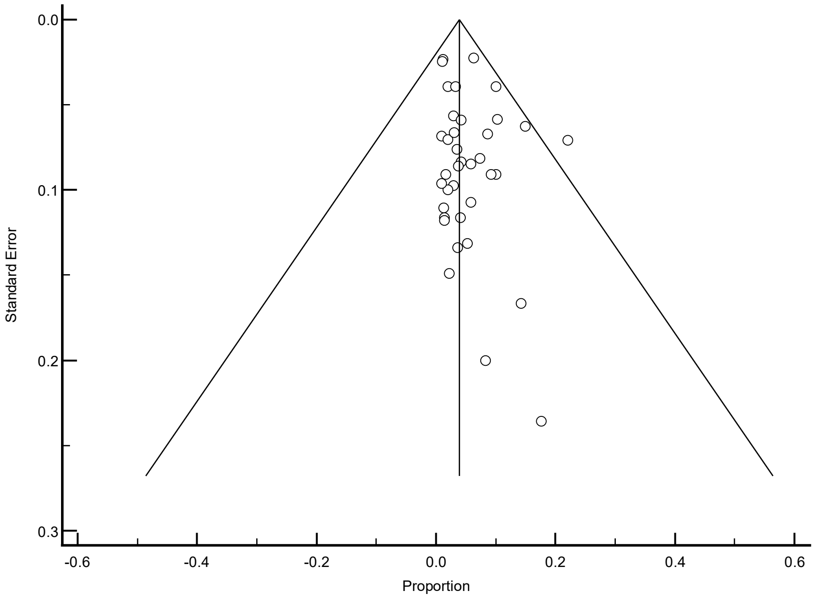

Temporary cement_Pulp necrosis

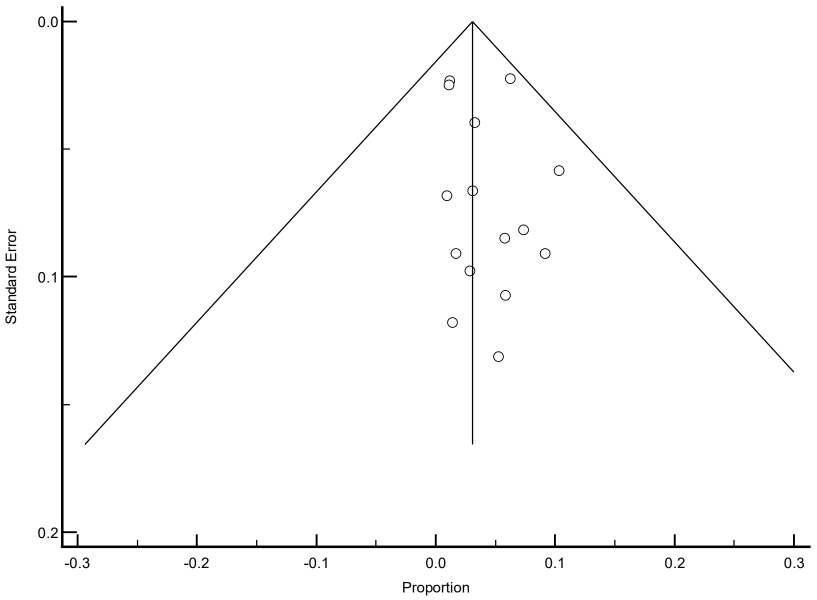

Temporization duration_Pulp necrosis

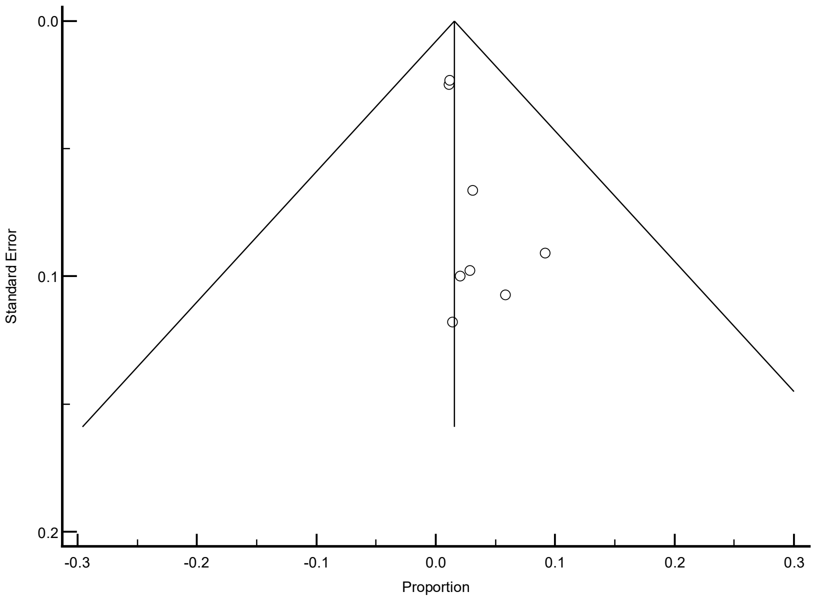

Impression material_Pulp necrosis

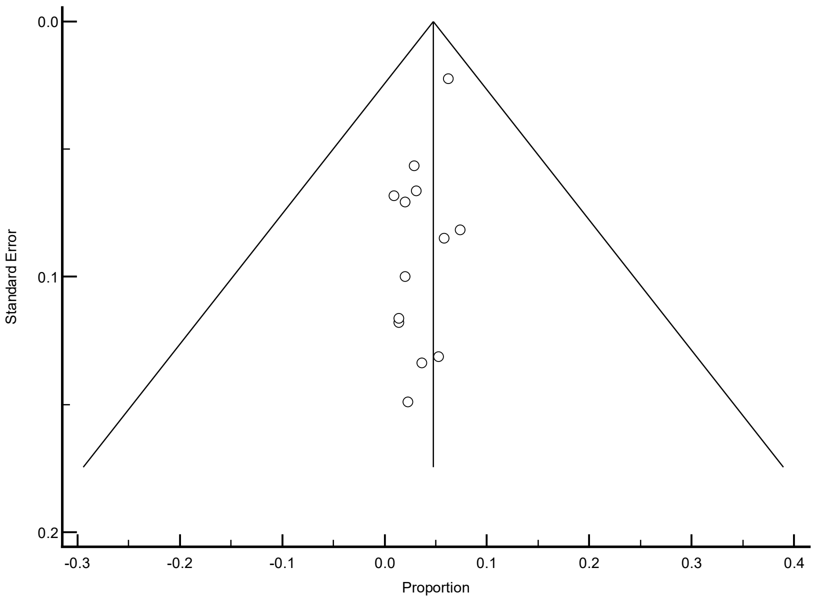

Restoration material_Pulp necrosis

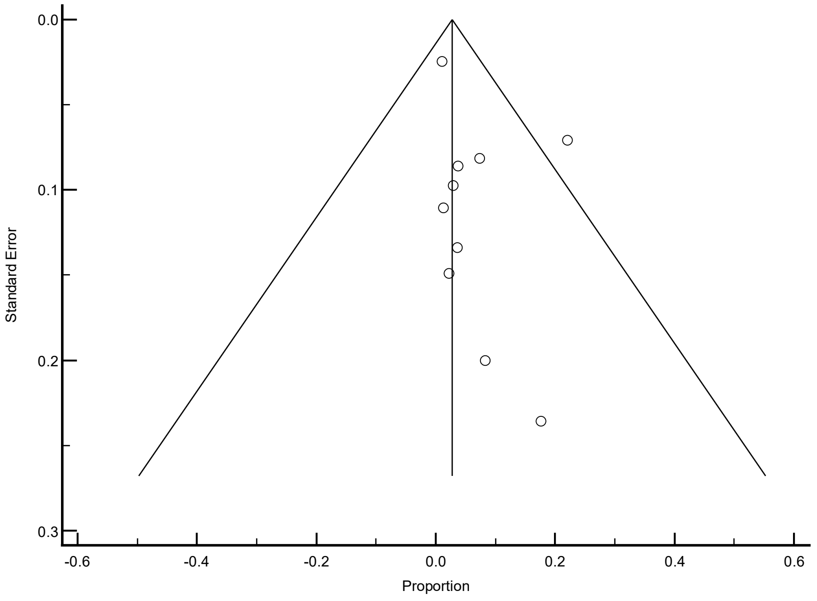

Permanent cement_Pulp necrosis

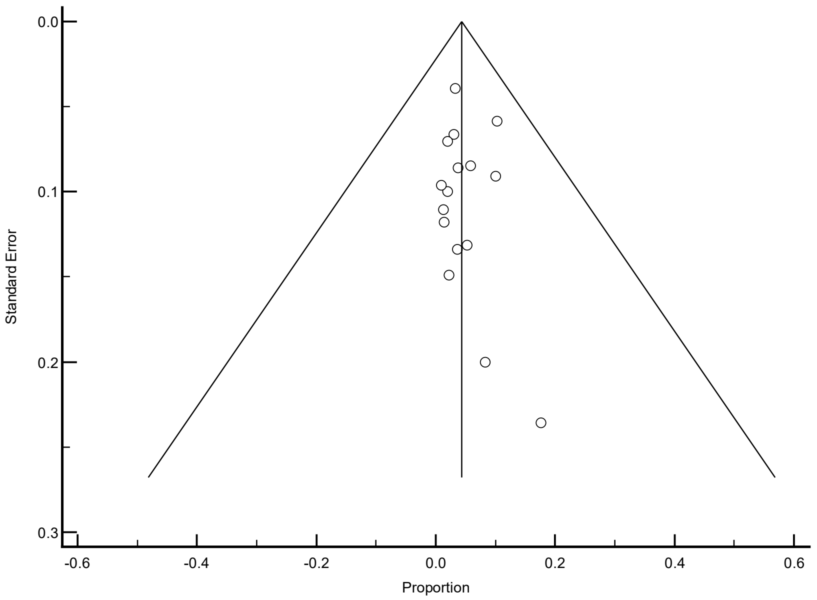

Posttreatment follow-up_Pulp necrosis

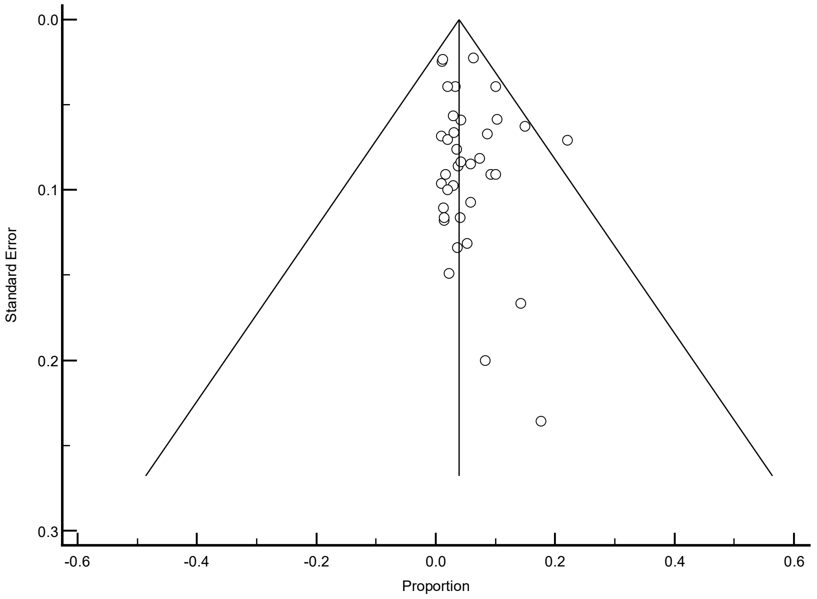

Posttreatment follow-up_Periapical pathosis

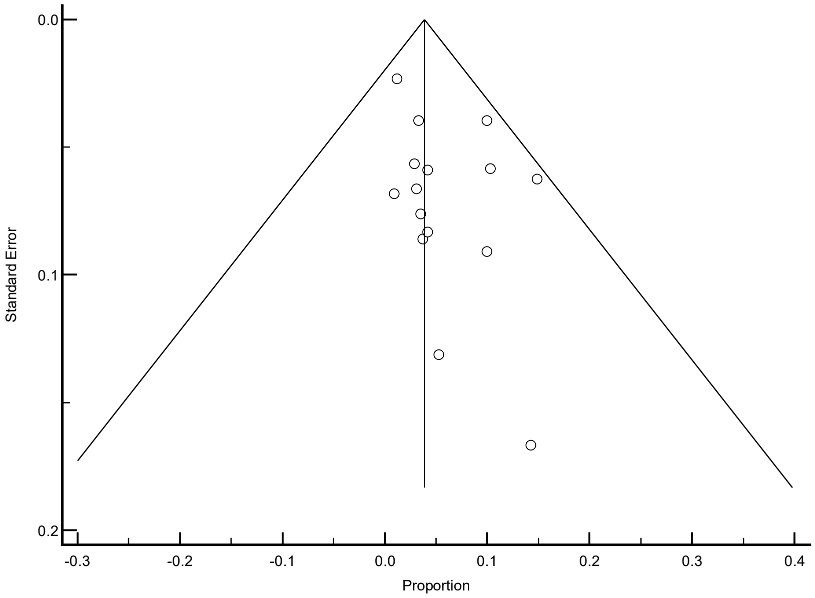

Clinical expertise_Pulp necrosis

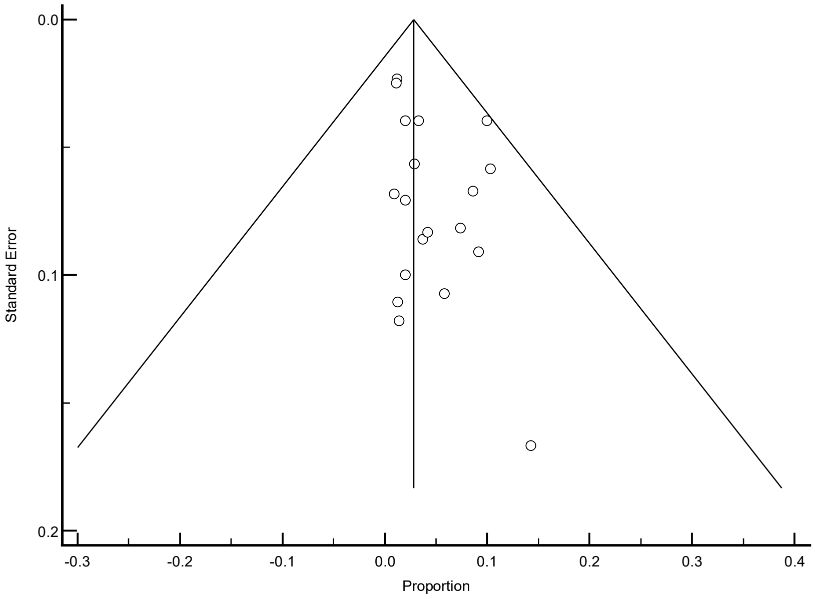

Supplement: Supplementary file 5 — Additional file 5: Supplementary file 5. Funnel plots. [file 12903_2023_2826_MOESM5_ESM.pdf]
