# Supplementary material for: Incidence and influential factors in pulp necrosis and periapical pathosis following indirect restorations: a systematic review and meta-analysis
Source: BMC Oral Health. 2023 Apr 2;23:195. doi: 10.1186/s12903-023-02826-1 (PMC10069144; doi:10.1186/s12903-023-02826-1)
Supplement: Supplementary file 6 — Additional file 6: Supplementary File 6. GRADE assessment. [file 12903_2023_2826_MOESM6_ESM.pdf]

Supplementary File 6: Pulp Necrosis Following Indirect Restorations: Grading of Recommendation, Assessment, Development and Evaluations (GRADE) Assessment.

**Outcomes**	**Certainty assessment**						
	**Studies' design (n)**	**Risk of bias**	**Inconsistency**	**Indirectness**	**Imprecision**	**Certainty**	**GRADE quality**
Overall incidence	Randomized trials (5) Observational studies (32)	Not serious	Serious	Serious	Not serious	⊕⊕◯◯ Low	Low
Type of indirect restorations	Randomized trials (4) Observational studies (28)	Not serious	Serious	Serious	Not serious	⊕⊕◯◯ Low	Low
Assessment method	Randomized trials (5) Observational studies (32)	Not serious	Serious	Serious	Not serious	⊕⊕◯◯ Low	Low
Type of temporary cement	Randomized trials (5) Observational studies (10)	Not serious	Serious	Serious	Not serious	⊕⊕◯◯ Low	Low
Duration of temporization	Randomized trials (1) Observational studies (7)	Not serious	Serious	Serious	Not serious	⊕⊕◯◯ Low	Low
Type of impression material	Randomized trials (4) Observational studies (9)	Not serious	Serious	Serious	Not serious	⊕⊕◯◯ Low	Low
Fabrication material	Randomized trials (1) Observational studies (9)	Not serious	Serious	Serious	Not serious	⊕⊕◯◯ Low	Low
Type of permanent cement	Randomized trials (2) Observational studies (14)	Not serious	Serious	Serious	Not serious	⊕⊕◯◯ Low	Low
Follow-up time	Randomized trials (5) Observational studies (32)	Not serious	Serious	Serious	Not serious	⊕⊕◯◯ Low	Low
Level of practitioner training	Randomized trials (2) Observational studies (17)	Not serious	Serious	Serious	Not serious	⊕⊕◯◯ Low	Low

Periapical Pathosis Following Indirect Restorations: Grading of Recommendation, Assessment, Development and Evaluations (GRADE) Assessment.

Outcomes	Certainty assessment						
	**Studies' design (n)**	**Risk of bias**	**Inconsistency**	**Indirectness**	**Imprecision**	**Certainty**	**GRADE quality**
Overall incidence	Randomized trials (2)<br>Observational studies (13)	Not serious	Serious	Serious	Not serious	⊕⊕◯◯<br>Low	Low
Type of indirect restorations	Randomized trials (2)<br>Observational studies (11)	Not serious	Serious	Serious	Not serious	⊕⊕◯◯<br>Low	Low
